# Supplementary material for: Measuring vaccination coverage and concerns of vaccine holdouts from web search logs
Source: Nat Commun. 2024 Aug 1;15:6496. doi: 10.1038/s41467-024-50614-4 (PMC11294546; doi:10.1038/s41467-024-50614-4)
Supplement: Supplementary file 1 — Supplementary Information [file 41467_2024_50614_MOESM1_ESM.pdf]

Supplementary Information for
Measuring Vaccination Coverage and Concerns of Vaccine Holdouts from Web Search Logs

Serina Chang[1,*], Adam Fourney[2], Eric Horvitz[2,†]

[1] Department of Computer Science, Stanford University
[2] Microsoft Research
[*] Research performed during an internship at Microsoft.
[†] Corresponding author. Email: horvitz@microsoft.com

In this Supplementary Information (SI), first we provide additional figures (S1-S16) and tables (S1-S7) that were mentioned in the main manuscript. Then, we provide supplementary analyses and discussion, figures, and tables.

State	Rank	URL
CA	0	https://myturn.ca.gov/
	1	https://www.cvs.com/immunizations/covid-19-vaccine
	2	https://www.goodrx.com/covid-19/walgreens
	3	https://www.costco.com/covid-vaccine.html
	4	https://www.walgreens.com/topic/promotion/covid-vaccine.jsp
NY	0	https://covid19vaccine.health.ny.gov/
	1	https://www.cvs.com/immunizations/covid-19-vaccine
	2	https://www.walgreens.com/topic/promotion/covid-vaccine.jsp
	3	https://vaccinefinder.nyc.gov/
	4	https://www.goodrx.com/covid-19/walgreens
TX	0	https://www.cvs.com/immunizations/covid-19-vaccine
	1	https://vaccine.heb.com/
	2	https://www.walgreens.com/topic/promotion/covid-vaccine.jsp
	3	https://corporate.walmart.com/covid-vaccine
	4	https://dshs.texas.gov/covidvaccine/
FL	0	https://www.publix.com/covid-vaccine
	1	https://www.cvs.com/immunizations/covid-19-vaccine
	2	https://www.walgreens.com/topic/promotion/covid-vaccine.jsp
	3	https://floridahealthcovid19.gov/vaccines/
	4	https://www.goodrx.com/covid-19/walgreens

Table S1: Results from S-PPR for California (CA), New York (NY), Texas (TX), and Florida (FL), the four largest states in the US. We display the top 5 URLs per state according to S-PPR scores. Note that there are common URLs across states, such as the CVS and Walgreens COVID-19 vaccine pages, but also state-specific programs in each set.

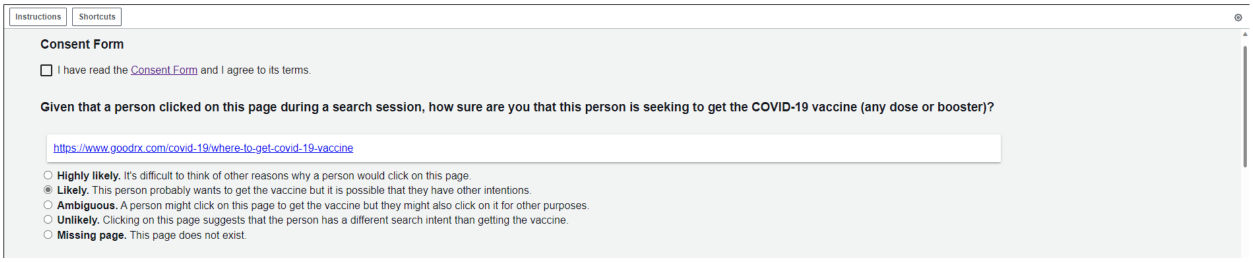

(a) First question.

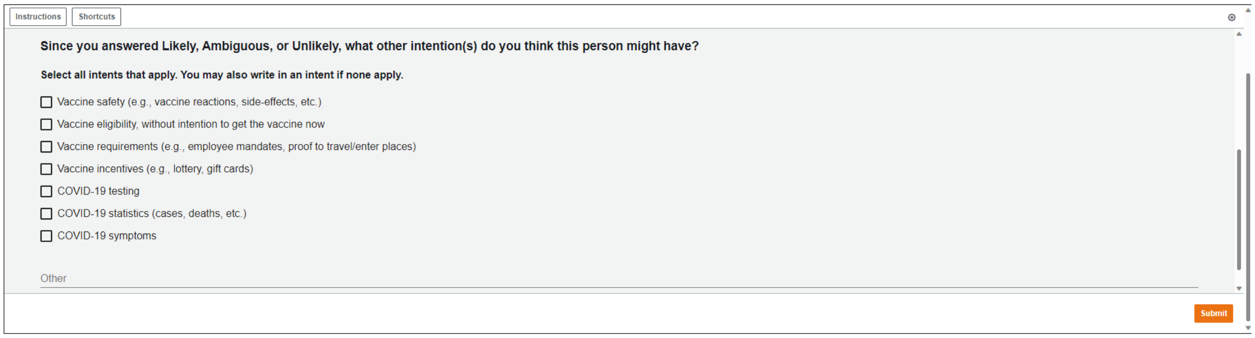

(b) Second question, which only shows if the annotator answers Likely, Ambiguous, or Unlikely to the first question.

Figure S1: Our Amazon Mechanical Turk (AMT) interface to collect vaccine intent annotations.

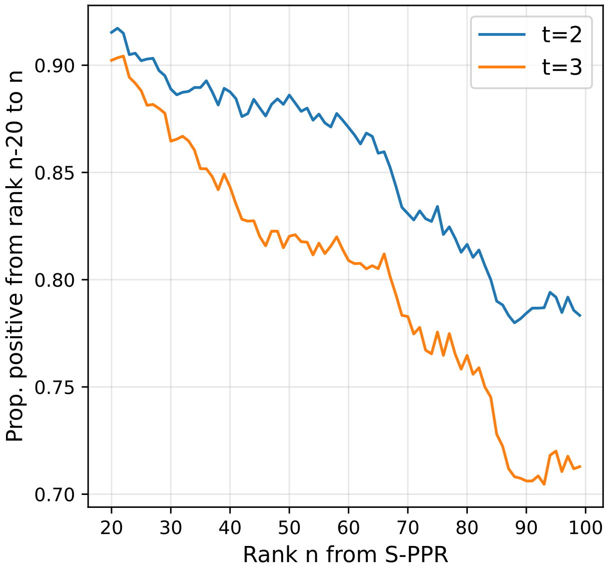

Figure S2: Comparison of S-PPR rank to the proportion of URLs around that rank that qualified for a positive vaccine intent label. We observe that these URLs (the top 100 from S-PPR) have a high positive rate overall: 86% if we require 3 positive annotations ($t = 3$), 90% if we require 2 ($t = 2$). Furthermore, the worse the S-PPR rank, the lower the positive rate, showing that our S-PPR technique is well-calibrated to predict vaccine intent.

State	Number of nodes	AUC w/o pre-train	AUC w/ pre-train
Wyoming	752865	0.741 (0.146)	0.951 (0.014)
Alaska	909357	0.796 (0.187)	0.921 (0.074)
Delaware	1269327	0.864 (0.134)	0.968 (0.007)
Montana	1533071	0.857 (0.139)	0.978 (0.011)
Connecticut	4407722	0.987 (0.005)	0.984 (0.008)
Tennessee	7712443	0.991 (0.003)	0.990 (0.003)

Table S2: Effects of pre-training on S-PPR rankings for 6 selected states that vary in size. We report the mean and standard deviation of AUC on the held-out test set over 10 random trials.

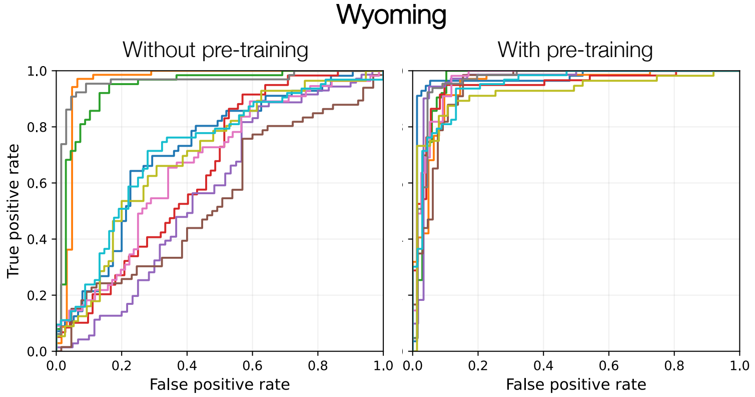

Figure S3: Visualizing the ROC curve for Wyoming, one of the smallest states. Each line represents a random trial. Without pre-training on S-PPR rankings, performance is unstable across trials, with some trials performing poorly (left). Pre-training stabilizes performance (right) and significantly improves AUC for smaller states (Table S2).

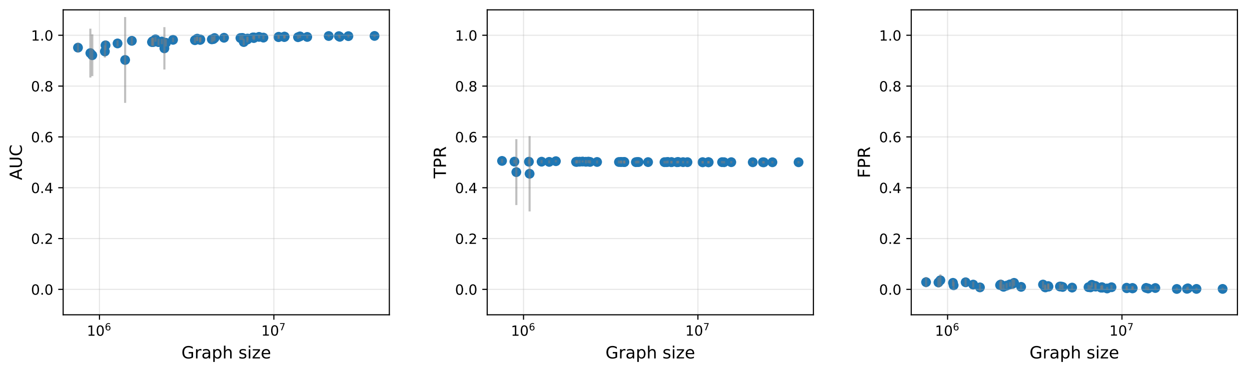

Figure S4: AUCs (left), true positive rates (middle), and false positive rates (right) across states. Metrics evaluate GNN performance on the held-out test set. Each dot represents a single state, with its y-coordinate representing the mean metric over 10 trials and grey bars indicating standard deviation.

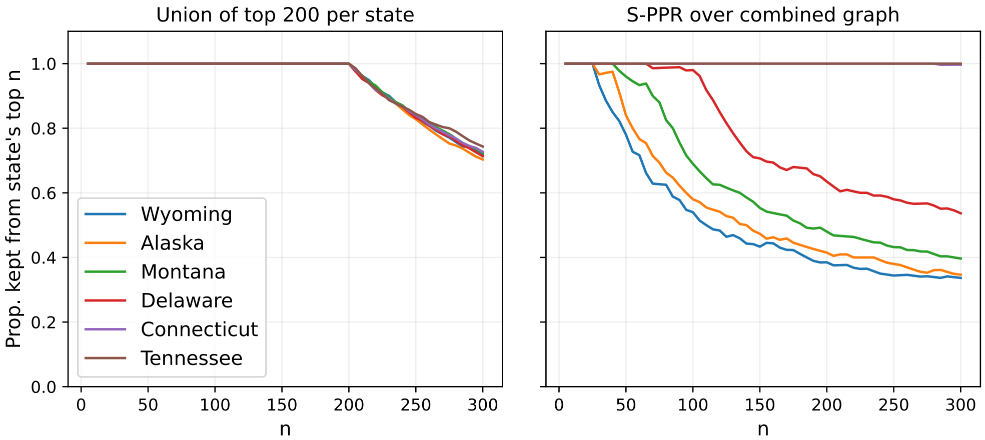

Figure S5: Comparing our union-over-states (left) to a combined graph approach (right) for generating URL candidates.

URL	t_{med}	t_{prec}
https://www.chesco.org/4836/61876/COVID-Authorized-Vax	7	10
https://patch.com/new-jersey/princeton/all-information-princeton-area-covid-vaccine-sites	9	10
https://dph.georgia.gov/locations/spalding-county-health-department-covid-vaccine	9	10
https://www.abc12.com/2021/04/22/whitmer-says-covid-19-vaccine-clinics-like-flint-church-are-key-to-meeting-goals/	7	10
https://www.delta.edu/coronavirus/covid-vaccine.html	10	10
https://www.lewistownsentinel.com/news/local-news/2021/01/scheduling-a-virus-vaccine-appointment/	9	10
https://www.laconiadailysun.com/news/local/covid-vaccine-clinics-at-lrgh-franklin-now-open-to-public/article_aa4b67e0-601a-11eb-a889-1bd4e6c83de1.html	6	10
https://www.insidenova.com/headlines/inside-woodbridges-new-mass-covid-19-vaccination-site-the-lines-keep-moving/article_eca45b88-8db0-11eb-a649-4bbeccd82cc3.html	9	10
https://www.keloland.com/news/healthbeat/coronavirus/avera-opens-covid-19-vaccine-clinic/	10	9
https://bangordailynews.com/2021/04/06/news/maine-to-kick-off-statewide-mobile-covid-19-vaccine-clinics-in-oxford-next-week-sk6sr8zcdk/	8	9
https://morgancounty.in.gov/covid-19-vaccinations/	9	10
https://www.firsthealth.org/specialties/more-services/covid-19-vaccine	10	10
https://healthonecares.com/covid-19/physician-practices/covid-19-vaccine-information.dot	9	10
https://patch.com/florida/stpete/drive-thru-covid-19-vaccine-sites-open-florida	9	10
https://vaccinate.iowa.gov/eligibility/	7	10
https://www.baynews9.com/fl/tampa/news/2021/03/17/new-walk-in-vaccine-site-at-tpepin-hospitality-centre-opens-today	10	10
https://www.doh.wa.gov/Emergencies/COVID19/VaccineInformation/FrequentlyAskedQuestions	10	10
https://www.emissourian.com/covid19/vaccine-registration-open-for-franklin-county/article_3638f7a0-5769-11eb-9bba-3f2611173784.html	10	10
https://www.fema.gov/press-release/20210223/maryland-open-covid-19-vaccination-center-waldorf-fema-support	10	10
https://kingcounty.gov/depts/health/covid-19/vaccine/forms.aspx	10	10

Table S3: A random sample (`random_state=0`) of 20 URLs from GNN. t_{med} and t_{prec} indicate how often the URL passed the median cutoff and precision cutoff, respectively, out of the 10 trials.

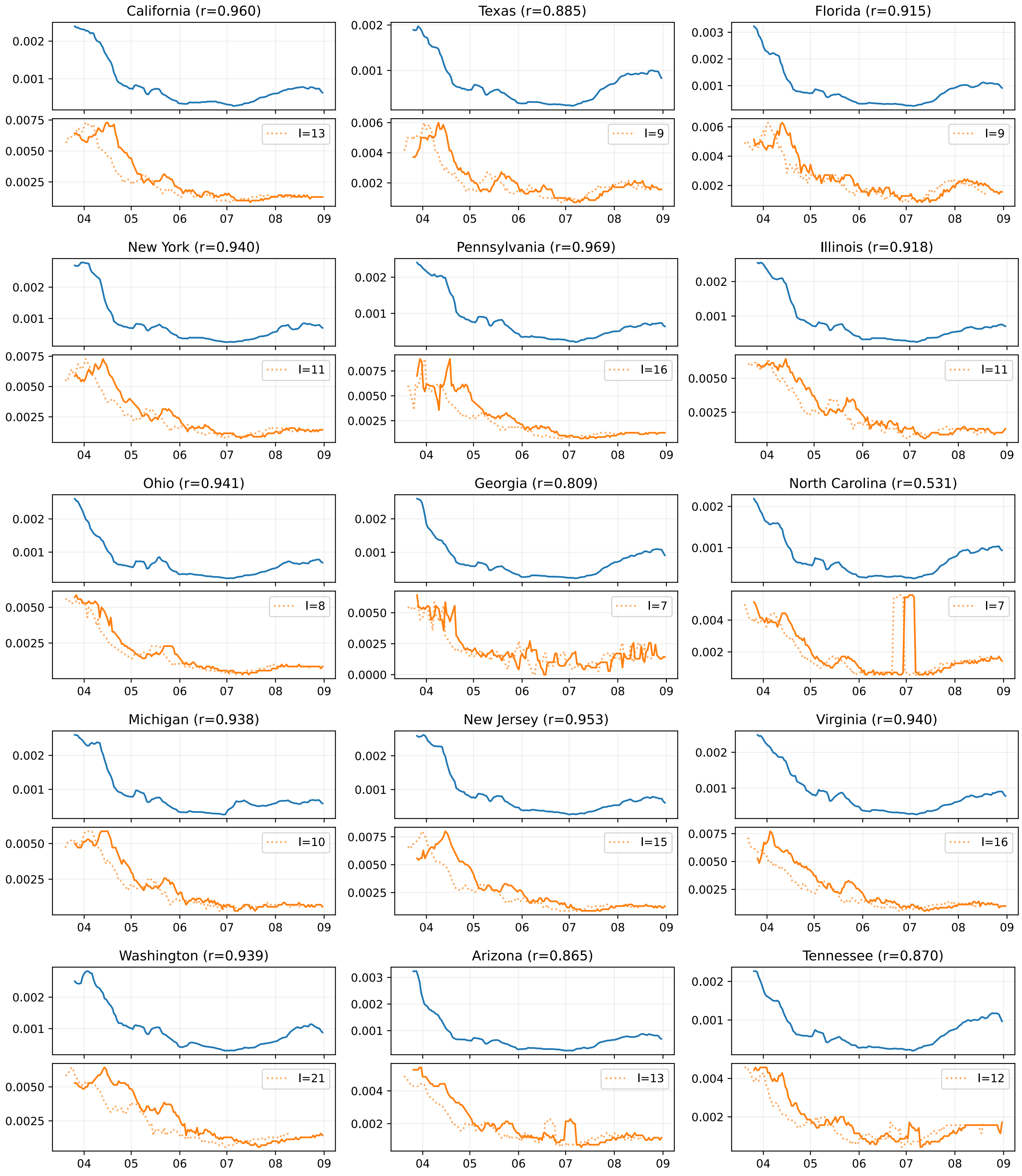

Figure S6: Comparison to CDC, states over time. Comparing the vaccine intent time series (top, blue) to the CDC vaccination time series (bottom, orange) for the 15 largest states in the US. We compute the maximum Pearson correlation between the time series (reported in each subfigure's title) when the CDC time series is allowed to lag by l days (reported in each legend). The dotted orange line in each bottom plot represents the adjusted CDC time series with the lag subtracted, so that the alignment between the time series can be more directly compared.

Lag (number of days)	SSR-based F Test	SSR-based Chi-Square Test	Likelihood ratio test
	Number of significant states ($p < 0.05$)		
7	37	43	40
14	26	36	32
21	24	36	33
28	25	42	39
	Number of significant states ($p < 0.01$)		
7	33	36	36
14	19	29	24
21	16	33	29
28	20	39	36

Table S4: Testing whether daily vaccine intent Granger-causes daily CDC vaccinations. We try four different lags and three different statistical tests: an F-test based on sum of squared residuals (SSR), a chi-squared test based on SSR, and a likelihood ratio test. For each combination, we report the number of US states where the test returns a significant result, for $p < 0.05$ (top) and $p < 0.01$ (bottom).

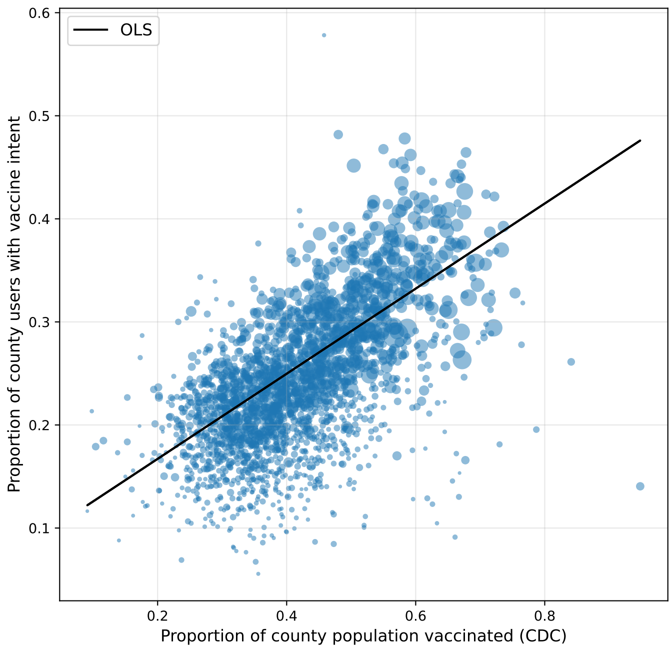

Figure S7: Comparison to CDC, counties. Comparing CDC county vaccination rates to estimated vaccine intent rates, cumulative up to August 31, 2021. The size of the dot and OLS fit are weighted by square root of county population.

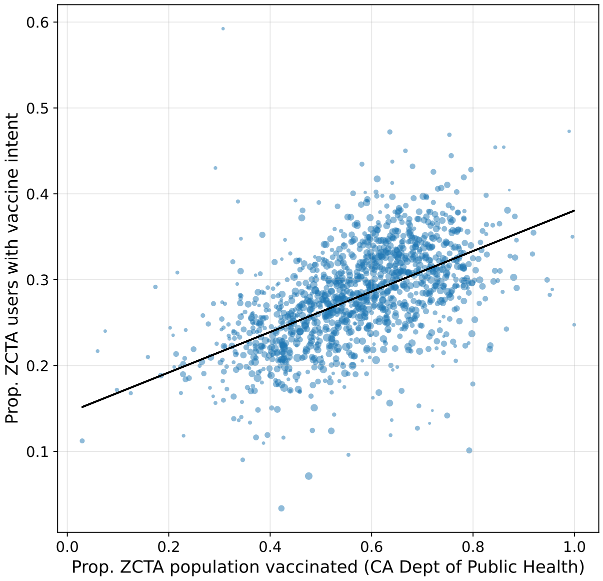

(a) ZCTA data from California Department of Public Health.

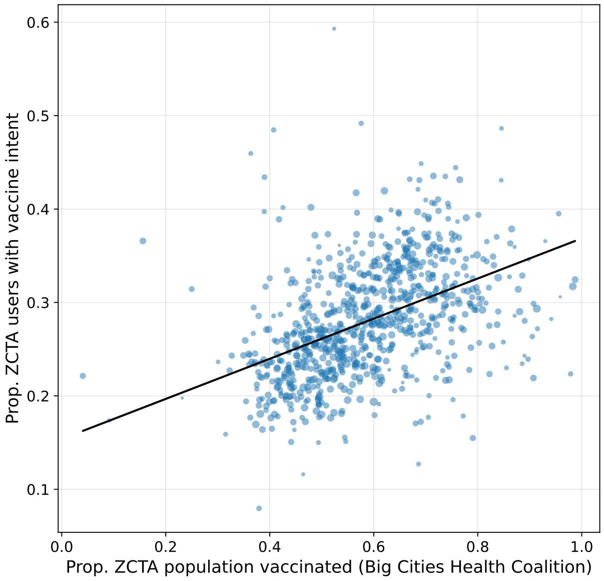

(b) ZCTA data from Big Cities Health Coalition.

Figure S8: Comparison to CDC, ZIP code tabulation areas (ZCTAs). Comparing ZCTA vaccination rates to estimated vaccine intent rates, cumulative up to August 31, 2021. The size of the dot and OLS fit are weighted by square root of ZCTA population.

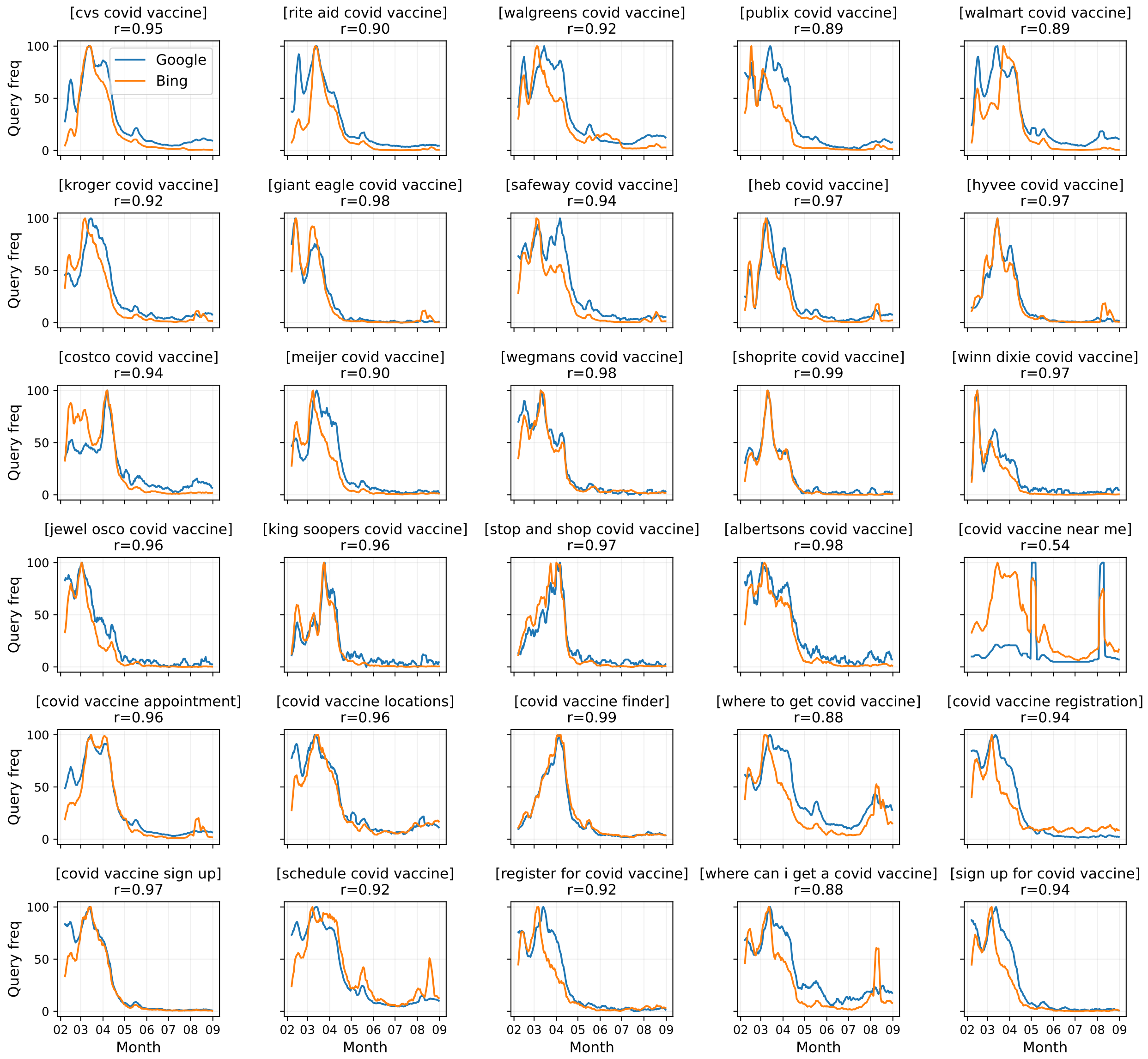

Figure S9: Comparing search trends over time on Google (blue) versus Bing (orange) for 30 of the most common vaccine intent queries. The y-axis represents query frequency, normalized so that 100 represents peak popularity in the US over the time period. Pearson correlations between normalized trends are reported in each subfigure's title.

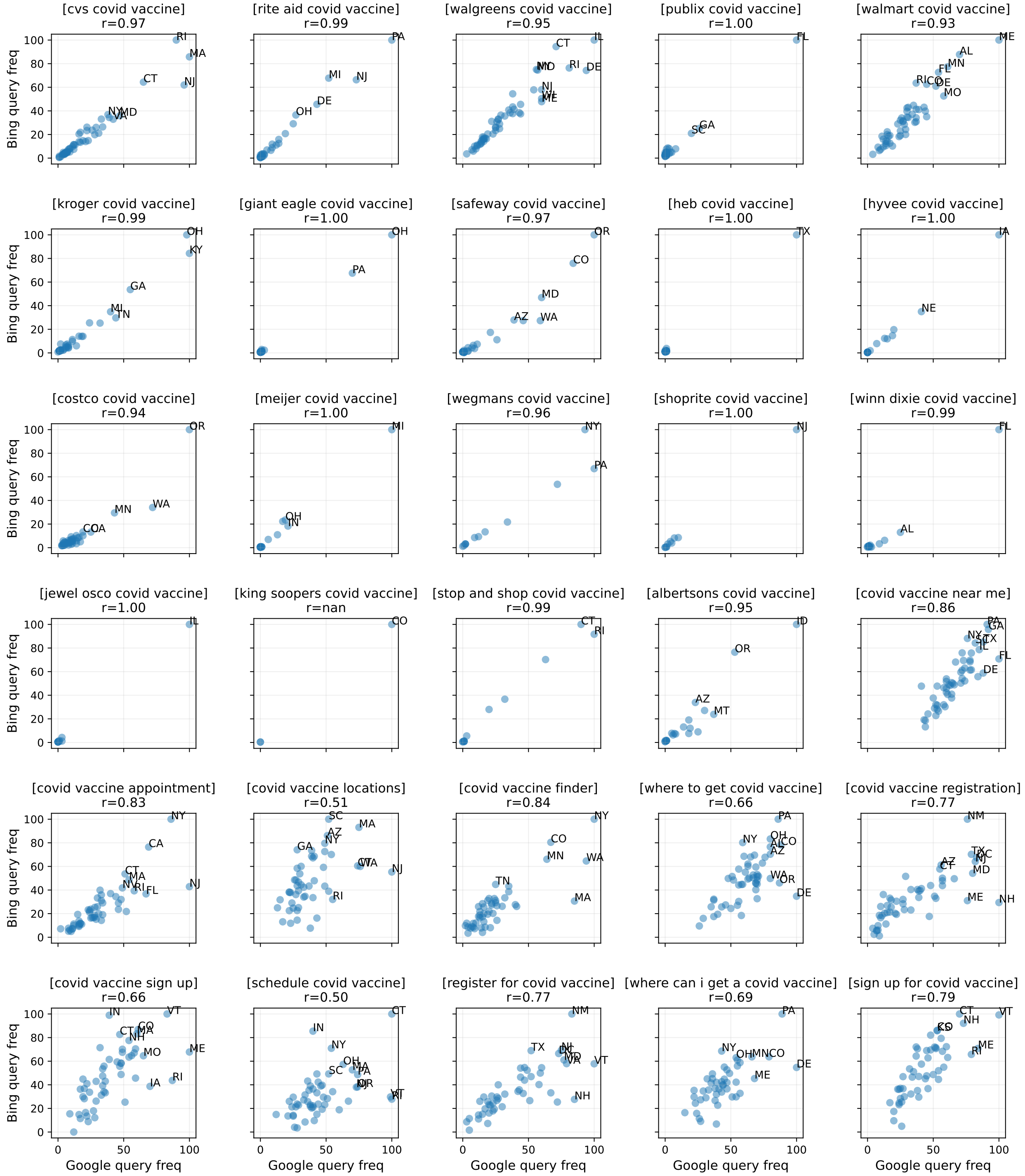

Figure S10: Comparing search trends across states on Google (x-axis) versus Bing (y-axis) for 30 of the most common vaccine intent queries. Following Google Trends, query frequency is measured as the fraction of the state's total queries that match this query, then normalized so that 100 corresponds to the maximum fraction over states.

# URLs	Top query	Top URLs	% Clicks
206	[cdc mask guidelines]	https://www.cbsnews.com/news/cdc-mask-guidelines-covid-vaccine	8.0
		https://www.cdc.gov/media/releases/2021/p0308-vaccinated-guidelines.html	6.9
		https://www.usatoday.com/story/news/health/2021/05/13/covid-vaccine-cdc-variant-fda-clots-world-health-organization/5066504001	4.5
		https://www.nytimes.com/2021/05/13/us/cdc-mask-guidelines-vaccinated.html	4.4
139	[vaers database covid-19]	https://www.cdc.gov/vaccinesafety/ensuringsafety/monitoring/vaers/index.html	17.0
		https://rightsfreedoms.wordpress.com/2021/07/22/vaers-whistleblower-45000-dead-from-covid-19-vaccines-within-3-days-of-vaccination-sparks-lawsuit-against-federal-government	6.8
		https://www.theburningplatform.com/2021/07/03/latest-cdc-vaers-data-show-reported-injuries-surpass-400000-following-covid-vaccines	5.7
		https://vaersanalysis.info/2021/08/20/vaers-summary-for-covid-19-vaccines-through-8-13-2021	4.9
137	[religious exemption for covid-19 vaccination]	https://www.verywellfamily.com/religious-exemptions-to-vaccines-2633702	16.5
		https://www.fisherphillips.com/news-insights/religious-objections-to-mandated-covid-19-vaccines-considerations-for-employers.html	5.1
		https://www.law360.com/articles/1312230/employers-should-plan-for-vaccine-religious-exemptions	3.9
		https://www.kxly.com/who-qualifies-for-a-religious-exemption-from-the-covid-19-vaccine	3.3
113	[johnson and johnson side effects]	https://www.openaccessgovernment.org/side-effects-johnson-johnson-vaccine/109505	20.3
		https://www.healthline.com/health/vaccinations/immunization-complications	8.1
		https://www.msn.com/en-us/health/medical/these-are-the-side-effects-from-the-johnson-and-johnson-covid-19-vaccine/ar-bb1f03fq	4.3
		https://www.healthline.com/health-news/mild-vs-severe-side-effects-from-the-johnson-and-johnson-covid-19-vaccine-what-to-know	4.3

Table S5: The 4 highest-modularity clusters with at least 100 URLs. For each cluster, we provide its number of URLs, its most frequent query, its top 4 URLs (by click frequency), and percentage of clicks over all clicks on URLs in the cluster that the URL accounts for.

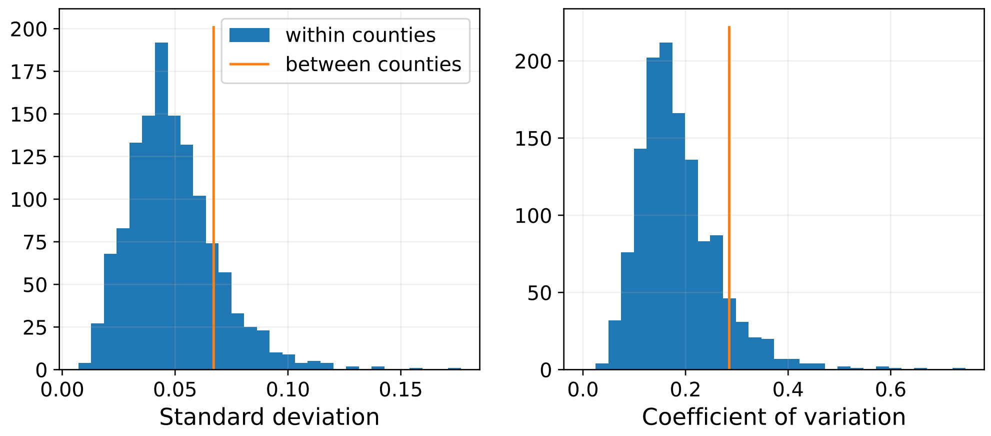

Figure S11: Comparing variation in estimated vaccine intent rates *within* counties (over ZCTAs) vs. *between* counties.

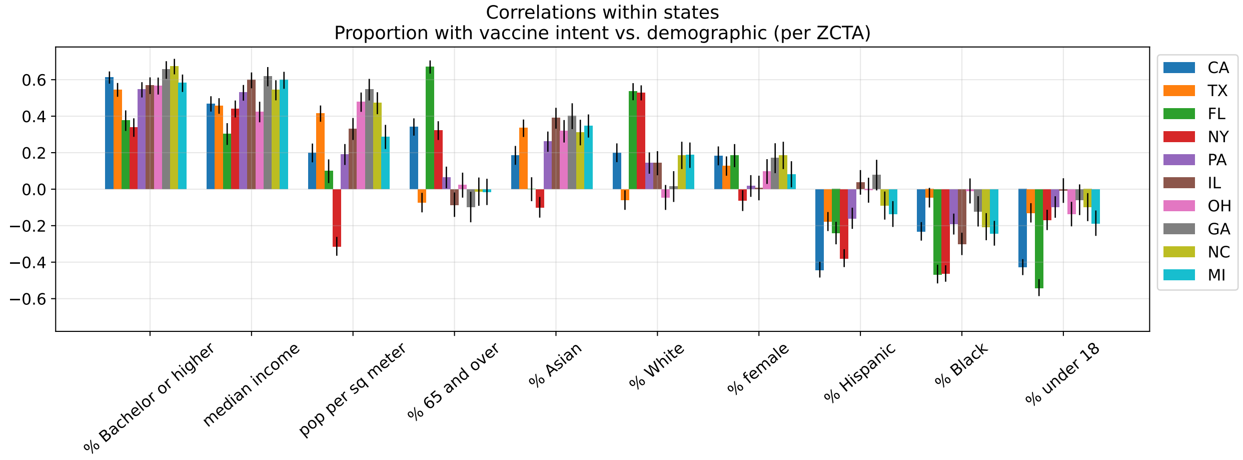

Figure S12: Demographic trends in vaccine intent. Comparing vaccine intent and demographics, for the 10 largest US states. Bar height indicates Pearson correlation between ZCTA vaccine intent rate and demographic variable, computed over $N = 1340$, 1328, 896, 1173, 1086, 846, 820, 540, 640, 741 ZCTAs per state (in order from from CA to MI). Error bars indicate 95% CIs.

Category	Subcategory	Ratio	2.5th pctl	97.5th pctl
Requirements	–	1.58	1.55	1.63
	Exemption	2.06	1.72	2.48
	Anti-mandate	2.00	1.93	2.09
	Fake proof	1.98	1.74	2.24
	Travel	1.40	1.34	1.47
	Proof	1.20	1.14	1.28
	Employment	1.16	1.02	1.30
Community	–	1.24	1.20	1.27
	Religious concerns	2.50	2.24	2.80
	Expert anti-vax	2.45	2.30	2.61
	Figure anti-vax	2.23	2.01	2.48
	News on hesitancy	1.20	1.13	1.26
	Vaccine rates	0.59	0.56	0.62
Safety	–	1.01	0.99	1.02
	Eerie fears	2.11	2.03	2.19
	FDA approval	1.72	1.58	1.87
	Vaccine-caused deaths	1.67	1.61	1.74
	Vaccine development	1.54	1.49	1.60
	Reproductive health	1.27	1.19	1.35
	Severe side effects	0.88	0.85	0.91
	Normal side effects	0.42	0.41	0.43
Effectiveness	–	0.84	0.82	0.87
	Natural immunity	1.30	1.21	1.39
	Efficacy against variants	1.24	1.13	1.37
	Breakthrough cases	1.10	1.05	1.16
	Efficacy from studies	0.57	0.55	0.60
Incentives	–	0.79	0.75	0.82
Information	–	0.72	0.71	0.74
	Decision-making	1.13	1.06	1.20
	Post-vax guidelines	1.10	1.05	1.14
	Special populations	0.87	0.81	0.94
	Johnson & Johnson	0.56	0.52	0.59
	Pfizer	0.53	0.50	0.57
	Comparison	0.53	0.48	0.57
	Moderna	0.37	0.35	0.39
Availability	–	0.22	0.21	0.24
	Boosters	0.78	0.65	0.91
	Children	0.70	0.64	0.76
	Locations	0.05	0.04	0.06

Table S6: Vaccine concerns of holdouts vs. matched early adopters. We report ratios of click probabilities within vaccine-related clicks from April to June 2021. 95% CIs are computed over 1000 bootstrapped samples. A higher ratio indicates a greater lean towards holdouts; a lower ratio indicates a greater lean towards early adopters. Categories, and subcategories within categories, are ordered from highest to lowest ratio.

Category	Subcategory	Ratio	2.5th pctl	97.5th pctl
Availability	–	4.68	4.49	4.88
	Locations	35.39	32.05	39.17
	Boosters	2.02	1.85	2.20
	Children	1.01	0.90	1.12
Incentives	–	2.46	2.30	2.61
Information	–	1.39	1.36	1.42
	Johnson & Johnson	4.29	4.07	4.54
	Comparison	2.52	2.33	2.72
	Moderna	1.20	1.10	1.29
	Special populations	1.19	1.08	1.30
	Pfizer	1.07	1.01	1.13
	Decision-making	1.06	0.99	1.14
	Post-vax guidelines	0.48	0.44	0.52
Safety	–	0.82	0.81	0.84
	Normal side effects	1.30	1.26	1.35
	Severe side effects	1.06	1.02	1.11
	Reproductive health	0.85	0.77	0.94
	FDA approval	0.75	0.70	0.81
	Vaccine development	0.68	0.64	0.71
	Eerie fears	0.64	0.61	0.68
	Vaccine-caused deaths	0.46	0.44	0.49
Effectiveness	–	0.80	0.77	0.83
	Efficacy from studies	1.20	1.13	1.28
	Efficacy against variants	0.76	0.71	0.82
	Natural immunity	0.67	0.62	0.72
	Breakthrough cases	0.58	0.54	0.62
Requirements	–	0.61	0.59	0.63
	Proof	1.02	0.96	1.08
	Fake proof	0.81	0.73	0.88
	Travel	0.64	0.57	0.72
	Employment	0.58	0.49	0.69
	Anti-mandate	0.52	0.49	0.54
	Exemption	0.36	0.33	0.40
Community	–	0.57	0.54	0.59
	News on hesitancy	0.86	0.80	0.92
	Vaccine rates	0.59	0.55	0.64
	Expert anti-vax	0.49	0.45	0.52
	Religious concerns	0.40	0.36	0.44
	Figure anti-vax	0.30	0.20	0.42

Table S7: Vaccine concerns of holdouts close to vaccine intent versus not. We report ratios of click probabilities within vaccine-related clicks from July to August 2021. 95% CIs are computed over 1000 bootstrapped samples. A higher ratio indicates elevated interest near vaccine intent; a lower ratio indicates reduced interest. Categories, and subcategories within categories, are ordered from highest to lowest ratio.

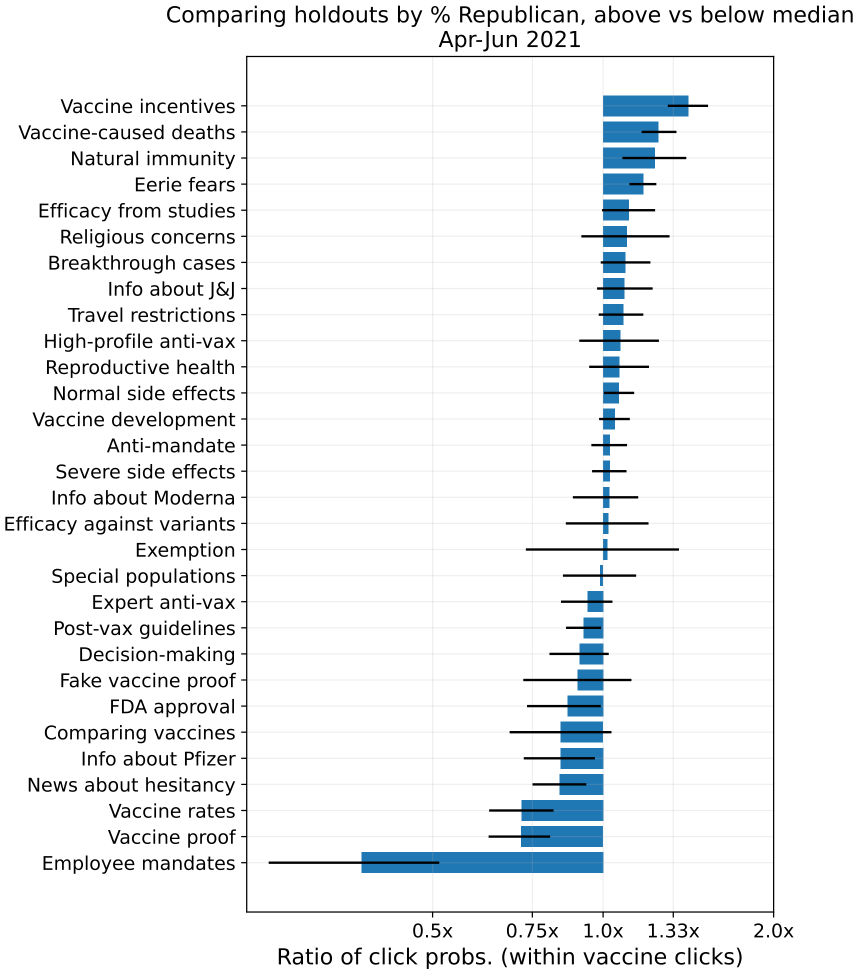

Figure S13: Comparing vaccine concerns of holdouts, split by those from counties above vs. below the median for percent Republican (based on 2020 presidential election). Error bars indicate 95% CIs computed over 1000 bootstrapped samples.

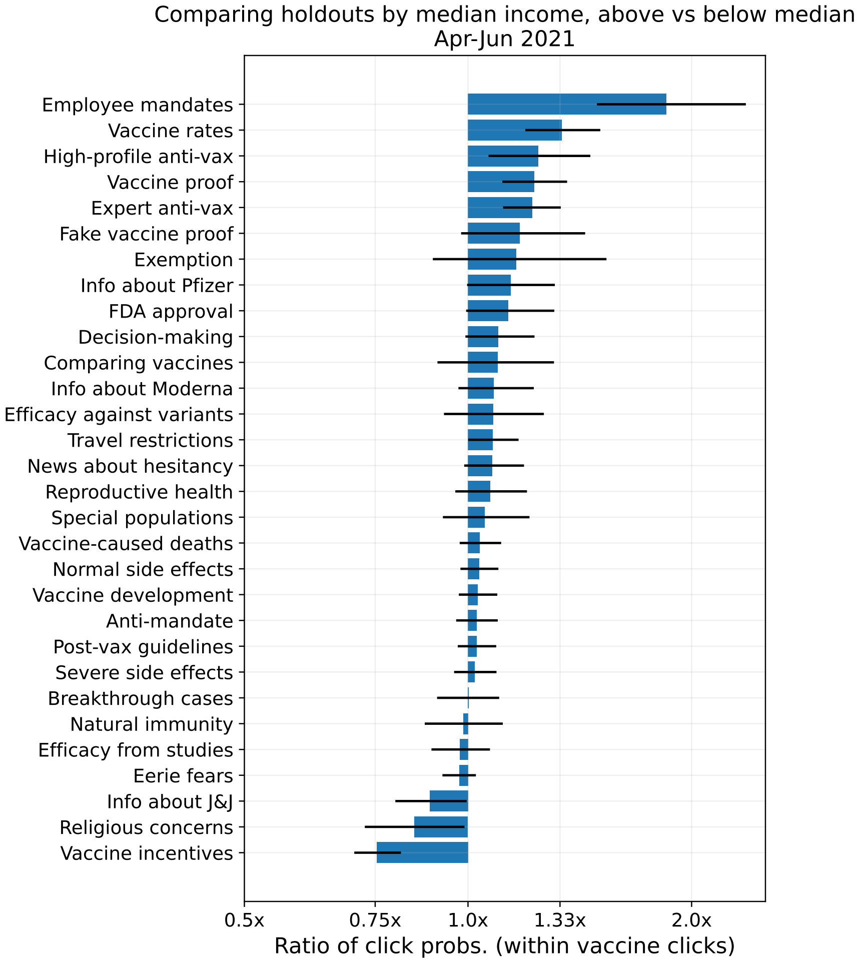

Figure S14: Comparing vaccine concerns of holdouts, split by those from ZCTAs above vs. below the median for median income. Error bars indicate 95% CIs computed over 1000 bootstrapped samples.

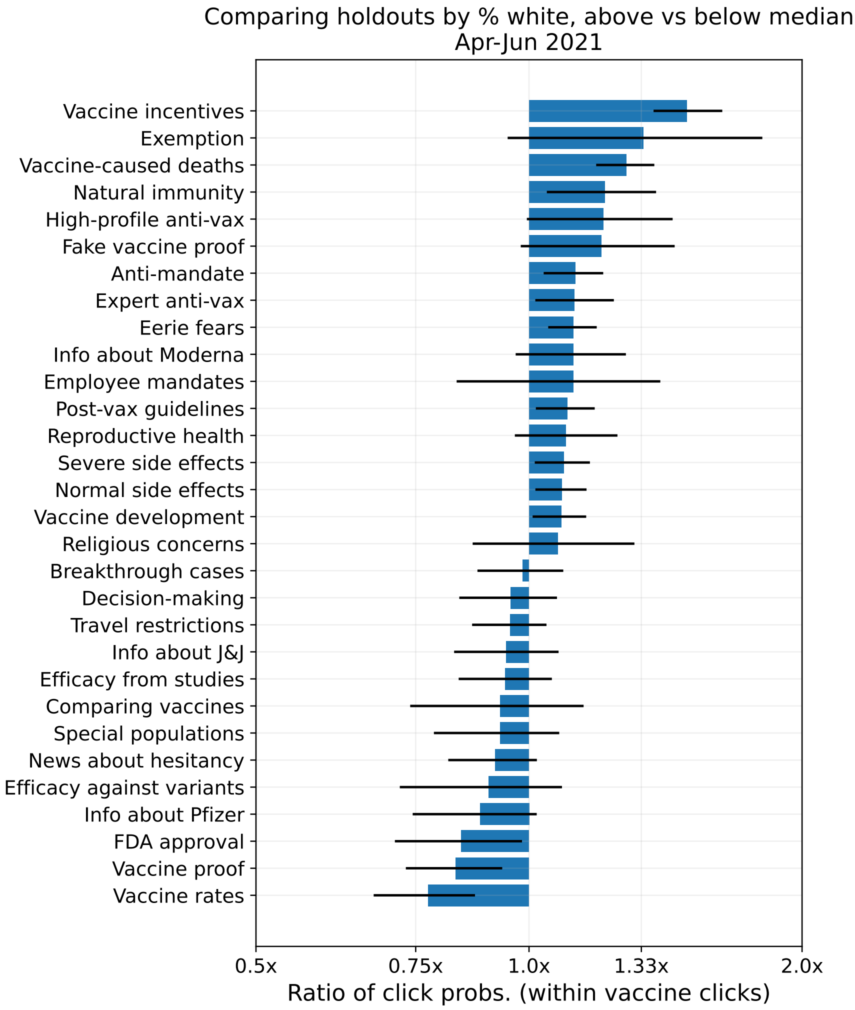

Figure S15: Comparing vaccine concerns of holdouts, split by those from ZCTAs above vs. below the median for percentage of residents who are white. Error bars indicate 95% CIs computed over 1000 bootstrapped samples.

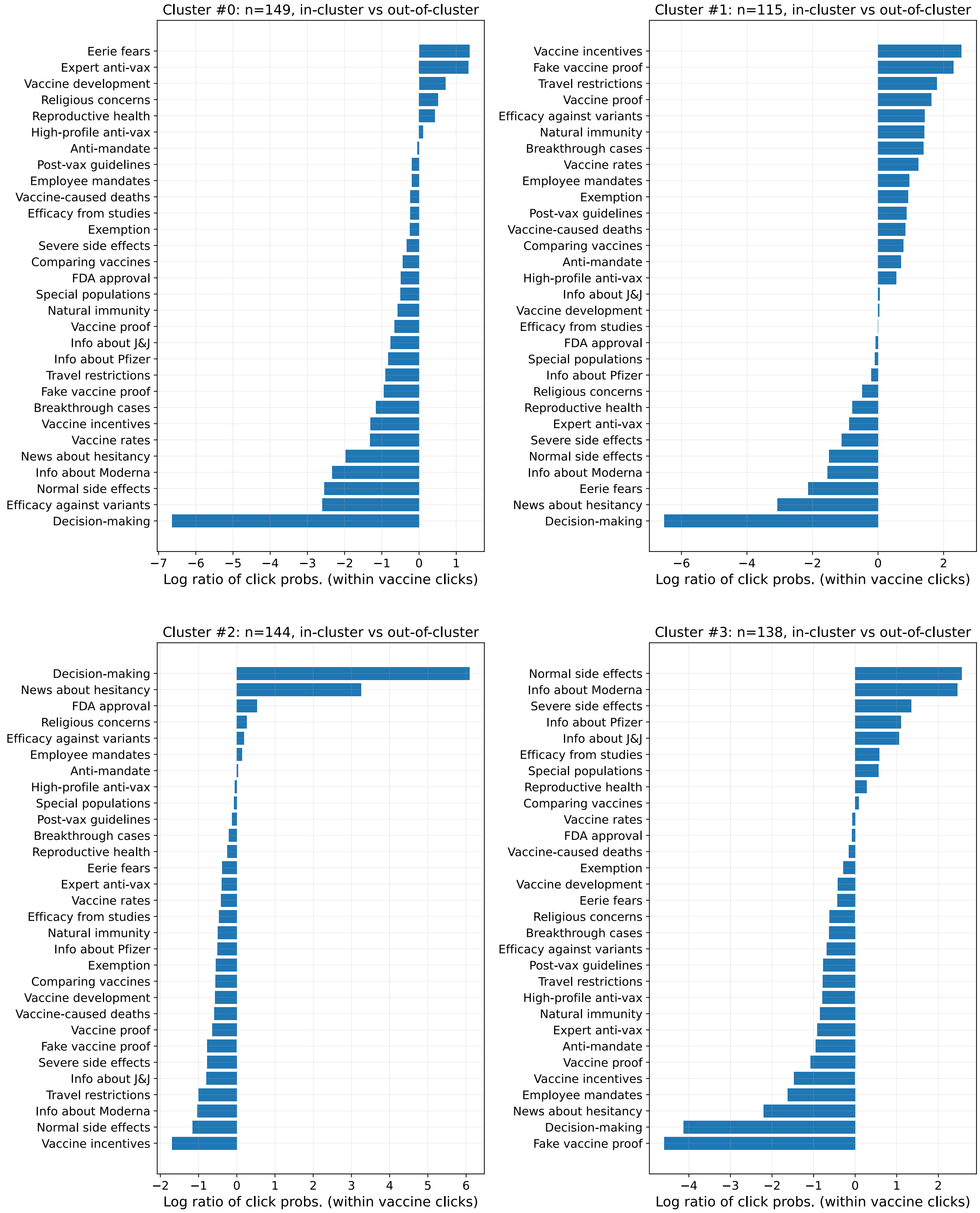

Figure S16: Holdout profiles. We discover four holdout profiles by clustering holdouts by their expressed vaccine concerns. For each cluster/profile, we visualize the ratio of average vaccine concerns of holdouts *in* the cluster vs. average vaccine concerns of holdouts *not* in the cluster.

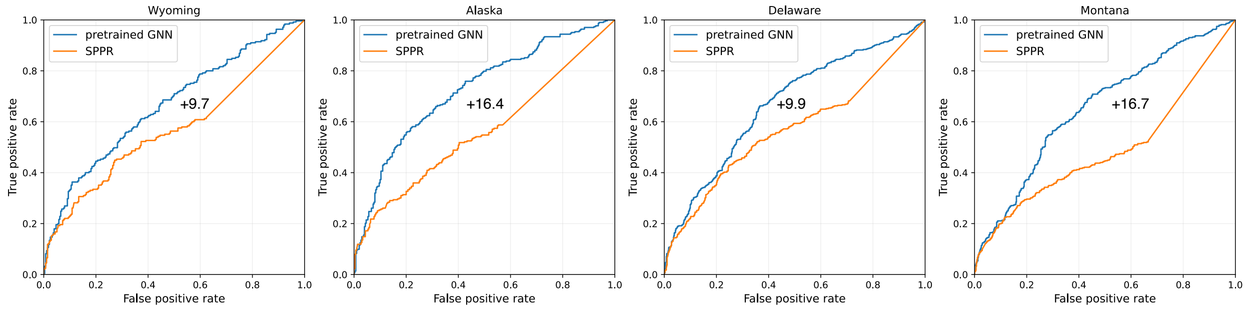

Figure S17: Comparing S-PPR vs. GNN pre-trained on S-PPR rankings (GNN-PPR). Despite only being trained on S-PPR rankings, GNN-PPR consistently outperforms S-PPR at predicting URL labels from AMT (unseen by both). In the figures, the number between the curves indicates the increase in AUC from S-PPR to GNN-PPR.

S1 Vaccine intent classifier: extended results and discussion

In this section, we provide supplementary experiments and discussion around our vaccine intent classifier. First, we describe an additional experiment where we show that, before training on *any* URL vaccine intent labels, the GNN pre-trained on S-PPR rankings already outperforms S-PPR at predicting URL labels (Section S1.1). Second, we provide two discussions: one about our design choice to include one graph per state (Section S1.2) and one about temporal drift in search signals (Section S1.3). Finally, we conduct an additional analysis of our estimated vaccine intent rates, where we characterize demographic trends by separating ZCTAs into top and bottom quartiles based on demographic variables (e.g., median income) (Section S1.4).

S1.1 Pretraining GNN on Personalized PageRank

As a supplementary experiment, we compare the predictive performance of S-PPR versus GNN-PPR, i.e., the GNN pre-trained on S-PPR rankings *before* it is also trained on AMT labels. Here, we evaluate on *all* AMT labels, since none of them were used in constructing S-PPR or GNN-PPR scores. In fact, evaluating on AMT labels is particularly challenging, since we chose to label only the top-ranked URLs according to S-PPR, so we are asking S-PPR to distinguish between URLs that it already considers similar. We conduct this experiment on the 26 smaller states for which we pre-trained our GNNs. First, we find across these states that S-PPR still performs better than random, with a mean AUC of 0.569, which complements our annotation results showing that even within its top-ranked URLs, S-PPR rankings still correlate with true rates of vaccine intent labels (Figure S2). Second, we find that GNN-PPR consistently *outperforms* S-PPR by 10-15 points, with a mean AUC of 0.675 (Figure S17). This is somewhat surprising, since GNN-PPR was only trained to predict S-PPR rankings, without any additional labels. We hypothesize that GNN-PPR outperforms S-PPR because, unlike S-PPR, the GNN can incorporate textual information from URLs and queries, in addition to graph structure. So, while S-PPR incorrectly upweights high-traffic URLs such as facebook.com that are often reached on random walks starting from the vaccine intent queries, GNN-PPR recognizes that these URLs do not look like the rest of high-ranking URLs and correctly excludes them. However, in order to achieve this difference between S-PPR and GNN-PPR, it is important not to overfit on S-PPR. So, we employ early stopping during pre-training; that is, we train the GNN on S-PPR rankings until they achieve a correlation of 0.8 and then we stop pre-training.

S1.2 Using a query-click graph per state

We considered using a "universal" graph of all 50 states, but a major constraint was space. The state-level graphs were already very large, e.g., half of the states have over 5 million nodes, and the largest state, California, has 38 million nodes and 111 million edges. Combining all 50 states into a "universal" graph would result in an extremely large graph, and it was in fact simpler computationally to train the GNN on each state than to attempt the universal graph. Furthermore, as we showed when we tried combining six states into one graph, larger states dominate the graph such that algorithms over the graph, like personalized PageRank, pay far more attention to their query-click patterns than that of smaller states, so smaller states' top-ranking URLs end up getting left out (Figure S5). One option would be to assign different weights to nodes or edges, e.g., upweighting query-click patterns for smaller states, but it is unclear what the right assignment is. For example, should we upweight to the extent that each state's query-click patterns contribute equally? That would result in miniscule weights for large states like California, and California genuinely has much more data so perhaps that is meaningful to record. Or, should we use the held-out validation set to tune what the weights should be, so that performance is similar for each state? However, performance per state within this universal graph approach is ill-defined, since performance is defined over URLs, which do not belong to a particular state. So, we did not pursue this direction in our current work due to the computational and algorithmic challenges, but this would be interesting to explore in future work.

S1.3 Discussion on temporal drift

While a benefit of search signals is that they appear in real-time and precede reporting, an important question is how to reap this benefit, since it requires time to validate the search signals against reported rates, and validity can change over time. We propose a two-step process: first, taking the time to develop the tool and evaluate it against reported rates over a substantial period of time, as we have in this work; second, deploying the tool but continually checking whether the tool's predicted signals continue to correlate with reported rates. During the second phase, it is possible to reap the benefits of search signals immediately (finer-grained and earlier signals), as long as the tool continues to correlate with reporting.

However, the validity of search tools may drift over time, which is why continuous validation is necessary. For example, Google Flu Trends (GFT),[1] which sought to predict flu prevalence from search queries, famously exhibited large errors after being released. There were several issues with their methodology, as discussed in Lazer et al.[2] First, GFT was prone to spurious correlations, since their system relied on finding queries (among 50 million possibilities) whose time series best matched reported flu trends; for example, GFT developers reported weeding out queries related to high school basketball. In comparison, our methodology only uses correlations for evaluation, not for constructing the classifier, and instead we rely on human expertise to construct it. Our regular expressions for labeling queries leverage domain knowledge about likely expressions of vaccine intent (e.g., requiring "covid", "vaccine", and a pharmacy name) and we acquire human annotations for URLs, making it highly unlikely for our classifier to pick up on non-vaccine-related queries or clicks. A second issue is that GFT is sensitive to Google's search algorithm and user behavior: for example, it overpredicted flu prevalence during a "media-stoked panic" during flu season and possibly also due to changes in Google's recommended searches.[2] Our system is less sensitive to such changes since we focus on precise vaccine intent, i.e., a user actively trying to

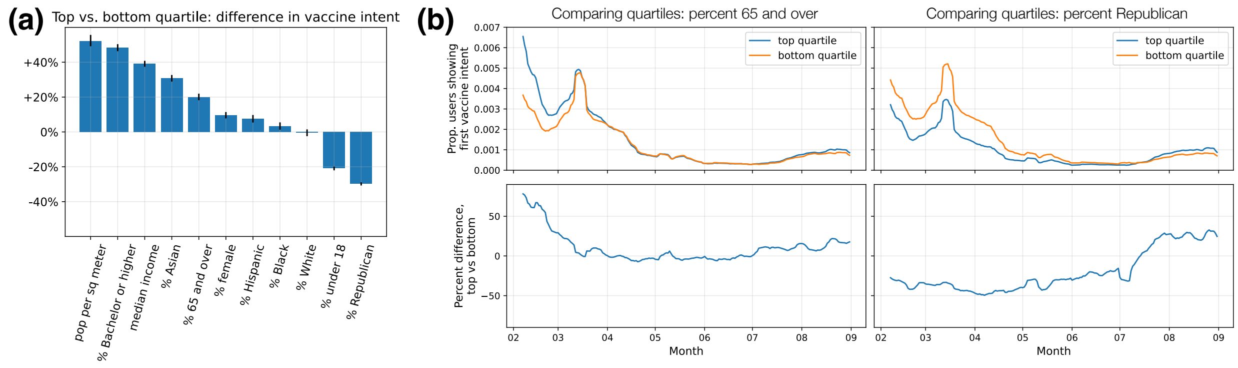

Figure S18: Demographic trends in vaccine intent, measured by quartile comparison. **(a)** We separate ZCTAs into top and bottom quartiles based on each demographic variable. Bar height indicates the percent difference in the top quartile's vs. bottom quartile's average vaccine intent rate. Error bars indicate 95% CIs over 1000 bootstrapped samples. **(b)** We can quantify changes over time in demographic trends by estimating average vaccine intent rates per quartile over time (top) and computing their percent difference (bottom).

get the COVID-19 vaccine on Bing, and rule out general interest in the vaccine (e.g., in eligibility or safety), and it is less likely that media trends or search recommendations would misrepresent a person's genuine vaccine intent as opposed to their general vaccine interest. Still, temporal drift is possible under our vaccine intent classifier; for example, if a new pharmacy begins offering the COVID-19 vaccine, we will need to add new queries and URLs related to that pharmacy to the classifier. An advantage of our classifier is that it is easily adaptable and does not require much additional annotation. For example, we could rerun personalized PageRank on an updated query-click graph and only check if any of the new top-ranking URLs are vaccine intent URLs that we should add, instead of needing to label a much larger set of URLs from scratch. Thus, our strategy for addressing temporal drift is two-fold: we develop a method that is both more robust to drift over time and easier to adapt to changes over time.

S1.4 Measuring vaccine intent demographic trends with quartiles

We also conduct a supplementary analysis where we use a different statistic to characterize demographic trends. We refer to this statistic as a "quartile comparison": we separate ZCTAs into top and bottom quartiles based on a demographic variable, then compare the quartiles' average vaccine intent rates (weighted by population size, following Eq. 5). Note that we compute quartile cutoffs based on *all* ZCTAs in the US Census, not only the ZCTAs that we are able to keep in our study. We compute bootstrapped CIs for these ratios by resampling the ZCTAs included (with replacement) then resampling each ZCTA's vaccine intent rate by sampling its number of users with vaccine intent from Binom($N(b, z)$, $\tilde{p}(v, z)$). We generate 1000 bootstrapped samples and, from the resulting distribution of ratios, report the 2.5th and 97.5th percentiles as the 95% CI. We find that demographic trends measured by quartile comparison (Figure S18a) largely agree with what we saw with correlations: population per square meter, percent with Bachelor degree, and median income continue to have the strongest positive relationships with vaccine intent, followed by percent Asian, percent 65 and over, and percent female. Percent Republican has the strongest negative relationship with vaccine intent, followed by percent under 18; the only differences with correlation are in percent White, Black, and Hispanic.

News domain	Ratio	Newsguard trust score
foxnews.com	1.23	69.5
nypost.com	1.11	69.5
dailymail.co.uk	1.04	64.5
forbes.com	1.04	100.0
huffpost.com	1.03	87.5
bbc.co.uk	1.01	95.0
businessinsider.com	1.00	100.0
theguardian.com	0.97	100.0
cnbc.com	0.92	95.0
wsj.com	0.92	100.0
usatoday.com	0.88	100.0
nbcnews.com	0.87	100.0
msn.com	0.87	100.0
news.yahoo.com	0.87	100.0
bbc.com	0.84	95.0
cnn.com	0.83	80.0
washingtonpost.com	0.81	100.0
nytimes.com	0.80	100.0

Table S8: Ratios and Newsguard trust scores for the most-visited news domains in the US. For each domain, we report the ratio of holdouts' vs. matched early adopters' probabilities of clicking on the news domain, using clicks from April to June 2021. Ratios above 1 indicate holdouts are likelier to click; ratios below 1 indicate early adopters are likelier to click. Domains are ordered from highest to lowest ratio.

Separating ZCTAs into top and bottom quartiles enables us to compute vaccine intent rates per quartile over time. We compute a time series per quartile in the same way that we computed the vaccine intent rate per state over time ("Comparison to reported vaccination rates"), by estimating the proportion of users who showed their *first* vaccine intent per day. Then, comparing the ratio of time series reveals changes in demographic trends over time (Figure S18b). For example, we estimate that older ZCTAs were much likelier to seek the vaccine early in 2021 but this trend fell over time, reflecting how the US vaccine rollout first prioritized seniors[3] then expanded to general eligibility,[4] and we see an increase in vaccine intent from more Republican ZCTAs in summer 2021, reflecting new calls from Republican leaders to get vaccinated[5] and a self-reported uptick in vaccinations among Republicans.[6]

S2 Holdout vs. early adopter analyses

In this section, we provide additional results from comparing vaccine holdouts and early adopters, matched on covariates. The extended analyses include results on news consumption (Section S2.1), reproductive concerns around the vaccine (Section S2.2), and the dynamics of vaccine concerns near conversion time (Section S2.3).

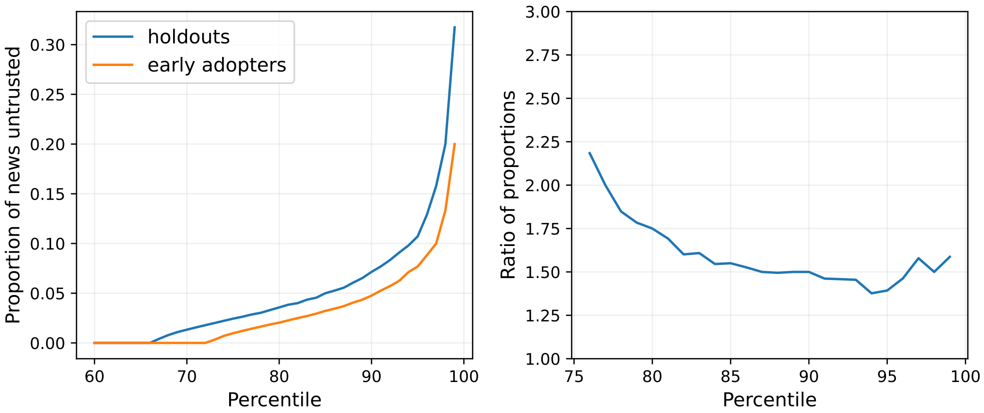

(a) Left: percentile plot, i.e., for holdouts and matched early adopters, we order them by their proportion of news untrusted and plot the proportion corresponding to the percentile. Right: for each percentile from 75th to 100th, we compute the ratio of the proportion of news untrusted for holdouts versus early adopters at that percentile.

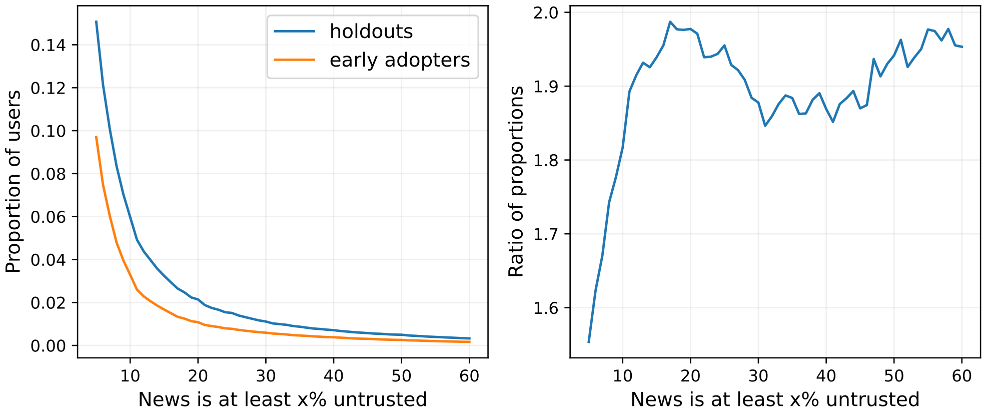

(b) Left: survival functions of proportion of news untrusted, for holdouts and early adopters. Right: ratio of survival functions, i.e., for each cutoff of x, the proportion of holdouts divided by the proportion of early adopters whose news is at least $x\%$ untrusted.

Figure S19: Comparing proportion of news untrusted. Proportion of news untrusted is measured as the proportion of an individual's news clicks that are on untrusted news sites. Here we compare holdouts and matched early adopters, using clicks from April to June 2021.

S2.1 Extended results on news consumption

In Table S8, we list the most-visited news sites in the US,[i] along with their holdout ratios and Newsguard trust scores. For example, holdouts are 23% likelier than matched early adopters to click on foxnews.com, with a trust score of 69.5, and 20% less likely to click on nytimes.com, with a trust score of 100.0. Notably, we are comparing holdouts to *matched* early adopters who come from the same county, which controls for regional political leaning. Without these controls, we would expect even larger gaps, given known associations between political leaning, vaccine adoption, and news trustworthiness.

In our main results, we showed that holdouts *on average* are far likelier (67%) to click on untrusted news than matched early adopters. However, to what extent is this result driven by a small number of holdouts clicking on a large amount of untrusted news, compared to holdouts

[i]https://www.statista.com/statistics/381569/leading-news-and-media-sites-usa-by-share-of-visits/.

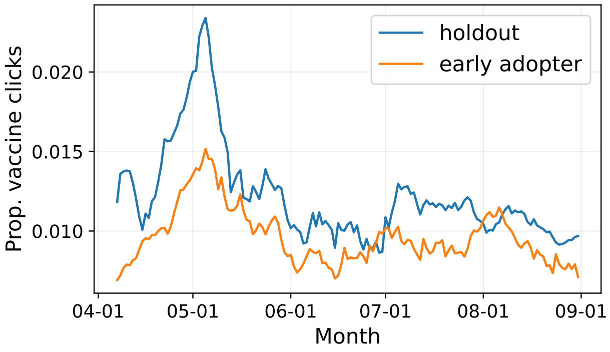

Figure S20: Interest over time in the Reproductive Health subcategory under Vaccine Safety.

broadly clicking on more untrusted news? To test this, we keep all holdouts and matched early adopters with at least 10 clicks on news, which leaves around 70% of each group (over 140,000 users in each). Then, for each user, we compute the proportion of their news clicks which are on untrusted news, and we make a percentile plot over users, where the x-axis is percentile rank and the y-axis is the proportion of news untrusted at that percentile (Figure S19a). If our average trend were, in fact, driven by a small number of holdouts, we should expect that the percentile curves for holdouts and early adopters are very similar until the final percentiles (e.g., above 95th), where the holdouts suddenly have much higher proportions. Instead, we find that the curves diverge as early as the 65th percentile, and throughout the final quartile (from 75th to 100th), holdouts—for the same percentile—consume $1.5\times$ to $2.25\times$ more untrusted news than early adopters. Thus, this plot reveals that it is not only a small number of holdouts driving the average trend. At the same time, from Figure S19a, we can see that even among holdouts, the majority do not consume any untrusted news at all. Furthermore, for the vast majority of holdouts (98%), untrusted news consists of less than 20% of their overall news consumption (Figure S19b). Representing the data in this way reveals that, while the elevated interest from holdouts in untrusted news is robust (not only driven by a small number of users), we also cannot assume that individuals are holding out only because of misinformation and false beliefs. This motivates the need for a more nuanced investigation of individuals' vaccine concerns, as we conducted in this work.

S2.2 Reproductive concerns around vaccine

Our taxonomy allows us to capture emergent information and interests. For example, we visualize relative interest in different top categories over time in Figure 4b, which reveals the appearance of vaccine incentives in mid-May 2021. Given the granularity of our taxonomy, we can also inspect time-varying interest in specific subcategories. In Figure S20, we visualize the proportion of vaccine-related clicks on the Reproductive Health subcategory under Vaccine Safety; this subcategory involves concerns about the vaccine's effect on fertility and the menstrual cycle. We observe a spike in interest in early May 2021, corresponding to stories at the time about the COVID vaccine affecting menstrual cycles, reported by sources such as ABC News[7] and Huffington Post[8]. Both holdouts and early adopters display spikes in interest at this time, although holdouts' spike is much larger, and they continue to be more interested in this subcategory than early adopters through the end of August 2021. There were also concerns throughout this time period about the vaccine's supposed negative effects on fertility, although scientists and doctors had been pushing back against

these concerns since January 2021[9]. Unfortunately, these myths became so widespread that by October 2021, nearly one third of US adults reported that "they heard that COVID-19 vaccines caused infertility and either believed the myth was true or weren't sure."[9]

S2.3 Dynamics of vaccine concerns near conversion time

In our main results, we showed that, when holdouts eventually express vaccine intent, their vaccine concerns nearly reverse such that they appear much more similar to early adopters than their typical selves (Figure 4d). Here, we conduct several supplemental analyses: (1) a robustness check where we control for time, (2) a more detailed version of the main analysis where we analyze each day relative to vaccine intent from -14 to +7 days, and (3) a *predictive* study that tests whether changes in vaccine concerns and news consumption Granger-cause vaccine intent.

Controlling for time. In our main analysis, we compared holdouts' vaccine concerns within a window of ± 3 days of vaccine intent versus outside of that window. However, the distribution of vaccine intent over this time period (July 1 to August 31, 2021) was not uniform; rather, vaccine intent became more likely over time. So, comparing concerns within the window of vaccine intent versus outside of the window could also capture changes in interests over time, separate from changes related to vaccine intent. In our main analysis, we estimate the probabilities that a vaccine-related click from a holdout is on a given subcategory when the holdout is in their vaccine intent window versus not, then compute the ratio of those probabilities. We can also estimate these probabilities using a logistic regression model, where we fit a model to predict whether a vaccine-related click from a holdout is on a given subcategory, conditioned on whether the holdout is in their vaccine intent window. Then, we can test the impact of controlling for time by extending the model to also condition on the date of the click. For a click x_i, let $v_i \in \{0, 1\}$ represent whether the click is within 3 days of the user expressing vaccine intent and let $t_i \in \{\text{2021/07/01}, \cdots, \text{2021/08/31}\}$ represent the date of the click. Then, for a given subcategory s, the full logistic regression model estimates the probability p_{is} that click x_i is on subcategory s as

$$p_{is} = \frac{1}{1 + \exp\left(-(\beta_s * v_i + \beta_{t_i,s})\right)}. \quad (1)$$

In other words, we learn subcategory-specific coefficients β_s for being in the vaccine intent window and $\beta_{t,s}$ for each day in the study period. We compare this model to a nested model where we replace $\beta_{t,s}$ with a single intercept β_0, so that we no longer control for time. Across all subcategories, we find that there is no significant change in β_s between the two models, so controlling for time does not have a significant impact. We also find that the learned β_s's are very similar to the ratios reported in Table S7, which is expected, since the β_s's represent log odds ratios and we report log probability ratios, and odds (i.e., $\frac{p}{1-p}$) and probability are similar when p is small.

Vaccine concerns per day near vaccine intent. We conduct a second analysis to deepen our understanding of the dynamics of vaccine concerns near vaccine intent. Previously, we compared click probabilities when a holdout was in their vaccine intent window (± 3 days) versus not in the window. Now, instead of only considering a binary variable, we consider a categorical variable k to represent the range of days from $k = -14$ days before vaccine intent to $k = +7$ days after. For each

day in that range, we estimate the probability of clicking on each vaccine subcategory (Figure S21). We can see that the probability of clicking on every subcategory is elevated close to vaccine intent, but some subcategories are much more elevated than others (e.g., comparing vaccines, Johnson & Johnson, vaccine incentives), which correspond to the most positive subcategories in Figure 4d. We can also see small differences in temporal trends. For example, interest in comparing vaccines and in the Johnson & Johnson vaccine becomes elevated well before vaccine intent, but interest in normal side effects only grows right before vaccine intent and stays elevated afterwards (perhaps reflecting individuals preparing for their vaccine appointment). Since all subcategories are elevated near vaccine intent but some more than others, we may want to normalize by the overall increase in vaccine-related interest to tease out differences between subcategories. As a secondary analysis, we estimate the probability of clicking on each subcategory per day, *conditioned* on the click being vaccine-related (which is analogous to our analysis in Figure 4d). Now, we can clearly see subcategories that have more elevated interest versus less elevated interest (Figure S22). Most notably, the bottom row—which represents the most holdout-leaning subcategories from Figure 4c—exhibits strong drops in relative interest as the individual approaches vaccine intent, especially in seeking exemptions to vaccine requirements, religious concerns about the vaccine, and anti-vaccine messages from high-profile figures and experts.

Predictive analysis of vaccine intent and Granger causality. So far, we have shown that the timing of vaccine intent is *associated* with changes in vaccine concerns, but are vaccine concerns actually *predictive* of vaccine intent? An affirmative answer here requires a higher bar than the previous two analyses for two reasons: first, we are only interested in changes in vaccine concerns that *precede* vaccine intent, instead of the entire window before and after vaccine intent; second, we are testing for predictive power beyond controlling for baseline predictors, including past values of vaccine intent and fixed day effects. These requirements can also be interpreted as testing for Granger causality (which is more rigorous than correlations but not yet causal): that is, we can interpret that vaccine concerns Granger-cause vaccine intent if our predictions of vaccine intent significantly improve when we include past values of vaccine concerns and vaccine intent in our model, compared to only including past values of vaccine intent.

For this analysis, we fit a series of nested logistic regression models. First, let $y_{ut} \in \{0, 1\}$ represent whether holdout user u expressed vaccine intent (either through queries or clicks) on day t. In a logistic regression model, the probability of $y_{ut} = 1$ is modeled as $1/(1 + e^{-\eta})$, where η is a linear function of observed covariates. Our baseline model $\mathcal{M}_{\text{base}}$ estimates η as

$$\eta_{\text{base}} = \sum_{l=1}^{L} \beta_l \cdot y_{u,t-l} + \beta_t, \tag{2}$$

where β_l is the coefficient on the l-th lag of vaccine intent, $y_{u,t-l}$, and β_t learns a fixed day effect for each day in the study period. We compare this baseline model to models that incorporate recent vaccine concerns as well as models that incorporate recent news consumption. First, we test two vaccine concern models: one model, $\mathcal{M}_{\text{vax}}$, that adds the user's number of clicks on any vaccine-related URL in the last week from $t-7$ to $t-1$ ($N^{\text{vax}}_{u,w(t)}$), and a second one, $\mathcal{M}_{\text{vax,sub}}$, that also adds their number of clicks on *each* relevant subcategory s ($N^{s}_{u,w(t)}$). To prevent leakage, we remove vaccine intent URLs from the counts in $N^{\text{vax}}_{u,w(t)}$, and remove all subcategories that might be related

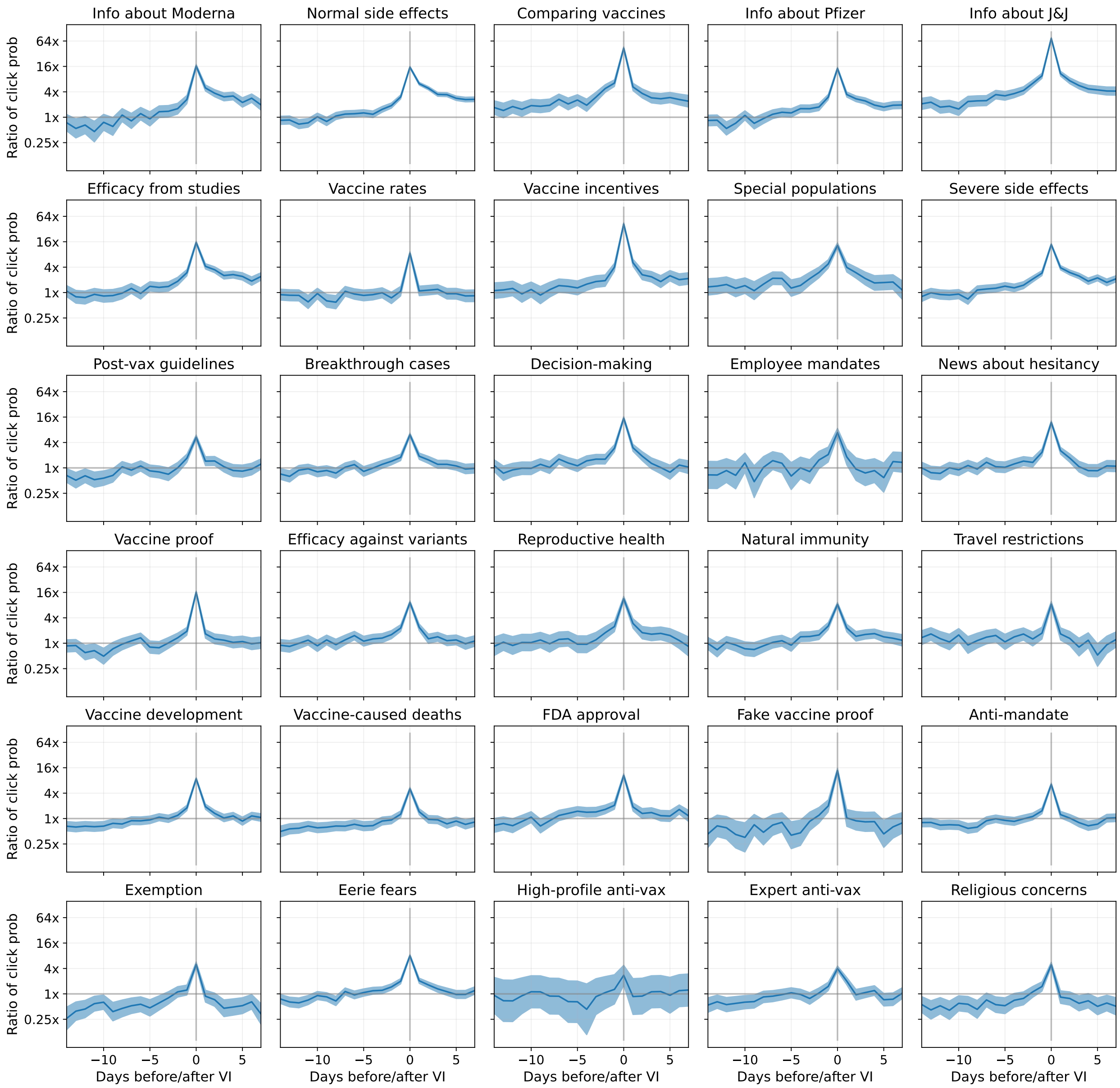

Figure S21: For each day from -14 days before to +7 days after vaccine intent, we measure holdouts' probability on clicking each subcategory. The blue line indicates that probability divided by their baseline probability outside of that 21-day range. Shaded regions represent 95% CIs computed over 100 bootstrapped samples. Subcategories are ordered from most early adopter-leaning to most holdout-leaning, according to Figure 4c. Interest in all subcategories is elevated near vaccine intent, but certain subcategories are much more elevated than others.

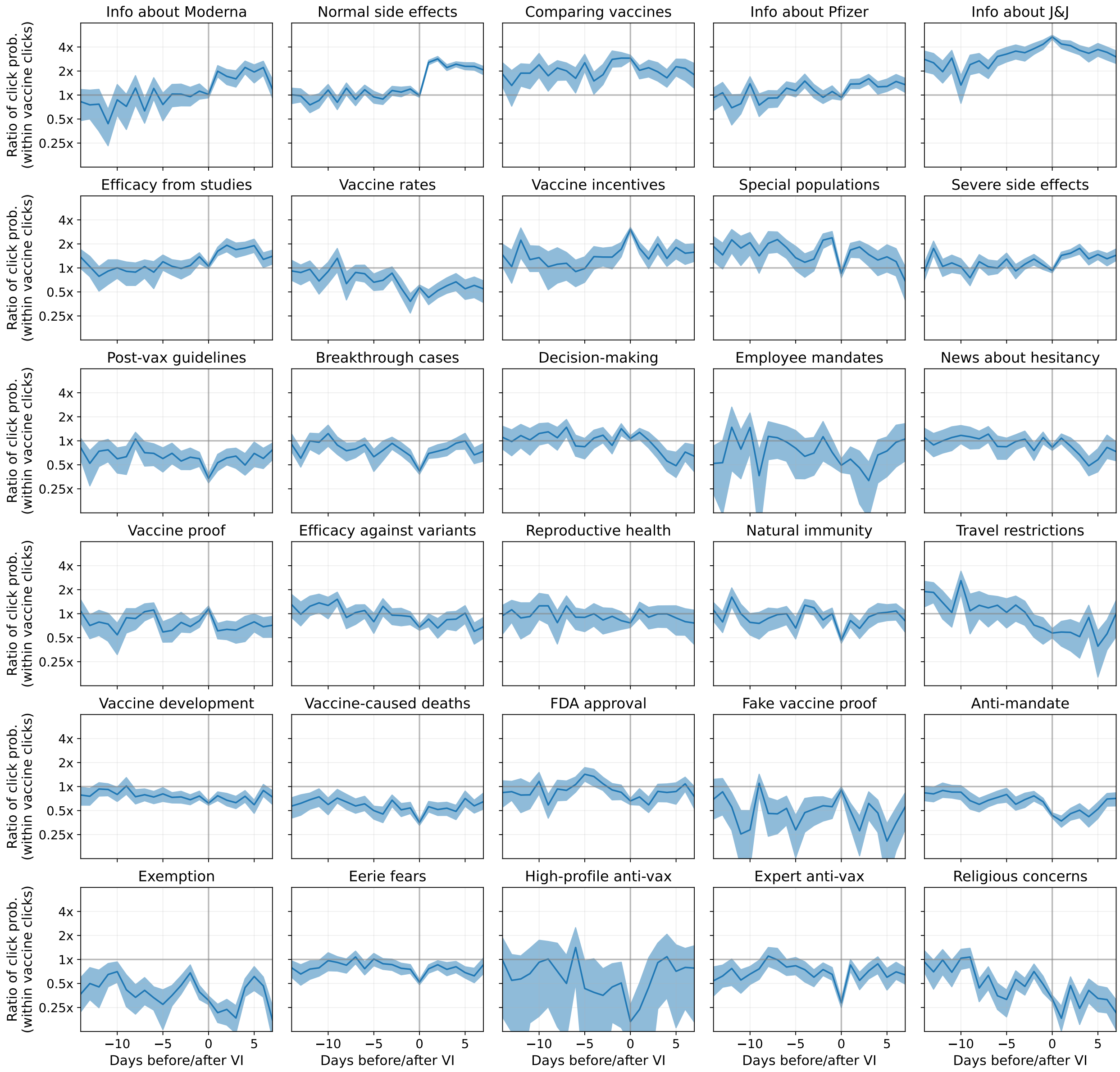

Figure S22: For each day from -14 days before to +7 days after vaccine intent, we measure holdouts' *relative* probability of clicking each subcategory (conditioned on the click being vaccine-related). The blue line indicates that probability divided by their baseline probability outside of that 21-day range. Shaded regions represent 95% CIs computed over 100 bootstrapped samples. Subcategories are ordered from most early adopter-leaning to most holdout-leaning, according to Figure 4c. Measuring within vaccine-related clicks emphasizes differences between subcategories.

to vaccine intent (all in the Availability category). Our vaccine concern models are defined as

$$\eta_{\text{vax}} = \sum_{l=1}^{L} \beta_l \cdot y_{u,t-l} + \beta_t + \beta_{vax} \cdot N_{u,w(t)}^{\text{vax}}, \tag{3}$$

$$\eta_{\text{vax,sub}} = \sum_{l=1}^{L} \beta_l \cdot y_{u,t-l} + \beta_t + \beta_{vax} \cdot N_{u,w(t)}^{\text{vax}} + \sum_{s} \beta_s \cdot N_{u,w(t)}^{s}. \tag{4}$$

We define two models here instead of only the latter since, from Figure S21, we saw that most vaccine subcategories are elevated near vaccine intent, so we want to see how much *additional* predictive power is gained from incorporating the specific subcategories clicked on, compared to overall interest in vaccines. Next, we test two news consumption models: one model, $\mathcal{M}_{\text{news}}$, that adds the user's number of clicks on any news in the last week ($N_{u,w(t)}^{\text{news}}$), and a second one, $\mathcal{M}_{\text{news,trust}}$, that also adds their number of clicks of trusted news ($N_{u,w(t)}^{\text{news,T}}$) and untrusted news ($N_{u,w(t)}^{\text{news,N}}$). Again, we remove vaccine intent URLs from these counts to prevent leakage. Our news consumption models are defined as

$$\eta_{\text{news}} = \sum_{l=1}^{L} \beta_l \cdot y_{u,t-l} + \beta_t + \beta_{\text{news}} \cdot N_{u,w(t)}^{\text{news}}, \tag{5}$$

$$\eta_{\text{news,trust}} = \sum_{l=1}^{L} \beta_l \cdot y_{u,t-l} + \beta_t + \beta_{\text{news}} \cdot N_{u,w(t)}^{\text{news}} + \beta_{\text{news,T}} \cdot N_{u,w(t)}^{\text{news,T}} + \beta_{\text{news,N}} \cdot N_{u,w(t)}^{\text{news,N}}. \tag{6}$$

Finally, our full model $\mathcal{M}_{\text{full}}$ uses the union of all features we tried in our previous models.

Using a likelihood ratio test for nested models, which accounts for the number of parameters added, we find that $\mathcal{M}_{\text{vax}}$ significantly outperforms $\mathcal{M}_{\text{base}}$ and $\mathcal{M}_{\text{vax,sub}}$ significantly outperforms $\mathcal{M}_{\text{vax}}$. Similar to our findings in the descriptive analyses, the vaccine subcategories with the most positive coefficients, β_s, are seeking information on the Johnson & Johnson vaccine, comparing vaccines, and decision-making (weighing the pros and cons of receiving the vaccine). We also find that $\mathcal{M}_{\text{news}}$ significantly outperforms $\mathcal{M}_{\text{base}}$ and $\mathcal{M}_{\text{news,trust}}$ significantly outperforms $\mathcal{M}_{\text{news}}$. Here, we find a significant positive coefficient on the amount of trusted news, $\beta_{\text{news,T}}$, while the coefficient on untrusted news, $\beta_{\text{news,N}}$ is not significant. Since the models with past values of vaccine concerns and news consumption outperform the baseline model, with past values of vaccine intent, we have shown that vaccine concerns and news consumption Granger-cause the timing of vaccine intent. Finally, we find that $\mathcal{M}_{\text{full}}$, which uses the union of all features, significantly outperforms both $\mathcal{M}_{\text{vax,sub}}$ and $\mathcal{M}_{\text{news,trust}}$, showing that vaccine concerns and news consumption provide complementary information. In all model comparisons reported here, we have $p < 10^{-29}$ from the likelihood ratio tests.

S3 Connection to Rogers' diffusion of innovations theory

Much of our analysis can be related to Everett Rogers' classic work on diffusion of innovations[10,11] and, specifically, his two frameworks for the decision-making process and adopter categories. First, we can map the five steps in his decision-making process to different points in the journey of vaccine adoption, with connections to specific categories in our taxonomy. We summarize this

Step	Name	Rogers' definition	Mapping to vaccine journey / our taxonomy
1	Knowledge / Awareness	The individual is first exposed to the innovation but lacks information about it	Learning about the vaccine (likely happened offline)
2	Persuasion	The individual is interested and actively seeks information about the innovation	Increased interest in Vaccine Safety, Vaccine Effectiveness, and Vaccine Requirements categories
3	Decision	The individual decides whether to adopt the innovation, weighing advantages / disadvantages	Decision-making subcategory (pros and cons of getting COVID-19 vaccine)
4	Implementation	The individual adopts the innovation	Planning vaccination (researching vaccine brands and expected side effects), booking vaccine appointment (vaccine intent), and receiving vaccination in-person
5	Confirmation / Continuation	The individual finalizes their decision to continue using the innovation	Boosters (not covered in this work)

Table S9: Mapping between Everett Rogers' decision-making process and vaccine adoption / our taxonomy.

mapping in Table S9, with further explanation below. Step 1, Knowledge / Awareness, occurs at the stage of learning about the vaccine, which likely happened offline and to most people, since the COVID-19 vaccine was widely discussed and anticipated. Step 2, Persuasion, may appear on search, and we see it through increased interest in the categories of Vaccine Safety, Vaccine Effectiveness, and Vaccine Requirements. Step 3, Decision, appears in the Decision-Making subcategory, where individuals are trying to understand the pros and cons of the COVID-19 vaccine. Step 4, Implementation, maps to subcategories where the individual is planning how they will receive the vaccine (e.g., researching the specific vaccine brands) and what to expect (e.g., side effects), along with booking their vaccine appointment (i.e., vaccine intent) and receiving the vaccine in-person. Step 5, Confirmation / Continuation, is less applicable in our setting, since within our study period (pre-booster), the vaccine is a one-time decision, but it would be interesting to use this final step to analyze who continues to seek out boosters.

Interestingly, when we cluster holdouts based on their vaccine concerns, we find that the clusters naturally align with these steps in the process (Figure S16). There is one cluster heavily emphasizing Vaccine Requirements and Vaccine Effectiveness (Step 2), one cluster emphasizing Decision-Making (Step 3), and one cluster emphasizing side effects and specific vaccine brands (Step 4). These clusters support the mapping between Rogers' theory and our taxonomy, and, by interpreting these concerns through Rogers' decision-making process, we can understand holdouts as varying significantly in their openness towards the vaccine and where they are in their conversion journey. Finally, as further evidence of the decision-making process, we saw in the prior analysis for predicting the timing of vaccine intent that the subcategories for decision-making, comparing

different vaccine brands, and interest in the Johnson & Johnson vaccine had the largest increases in interest in the week preceding vaccine intent, which correspond to Steps 3 and 4.

Rogers' adopter categorization defines five groups of adopters, based on their order of adoption: Innovators (first 2.5%), Early Adopters (13.5%), Early Majority (34%), Late Majority (34%), and Laggards (16%). In our work, we defined our "early adopters" as those who showed their vaccine intent before May 31, 2021, and our "holdouts" as those who showed their vaccine intent between July 1 and August 31, 2021. 50% of the US population received at least one dose by May 31, 2021, 55% by July 1, 62% by August 31, and 81% by May 2023.[12] This time series maps our "early adopters" to Rogers' Early Adopters and Early Majority classes and our "holdouts" to Rogers' Late Majority. Compared to the earlier adopters, Rogers theorizes that the Late Majority tend to be a skeptical group and cautious about innovation, who may adopt out of economic necessity or due to increasing social pressure.[11] We see evidence of such skepticism in the holdouts' vaccine concerns, compared to matched early adopters (Figure 4c). For example, holdouts are far more interested in how the vaccine was developed (with skepticism about the speed of development) and FDA approval. They also show heightened interest in vaccine requirements, such as mandates and required proof of vaccine, reflecting economic necessity and social pressure that possibly persuaded them to eventually adopt the vaccine.

References

1. Jeremy Ginsberg, Matthew H. Mohebbi, Rajan S. Patel, Lynnette Brammer, Mark S. Smolinski, and Larry Brilliant. Detecting influenza epidemics using search engine query data. *Nature*, 457:1012–1014, 2009.

2. David Lazer, Ryan Kennedy, Gary King, and Alessandro Vespignani. The parable of google flu: Traps in big data analysis. *Science*, 343:1203–1205, 2014.

3. Berkeley Lovelace Jr. Cdc expands covid vaccination guidelines to everyone 65 and older. *CNBC*, 2021. https://www.cnbc.com/2021/01/12/covid-vaccine-trump-administration-to-expand-eligibility-to-everyone-65-and-older.html.

4. Emily Anthes, Madeleine Ngo, and Eileen Sullivan. Adults in all u.s. states are now eligible for vaccination, hitting biden's target. half have had at least one dose. *The New York Times*, 2021. https://www.nytimes.com/2021/04/19/world/adults-eligible-covid-vaccine.html.

5. Marianna Sotomayor, Jacqueline Alemany, and Mike DeBonis. Growing number of republicans urge vaccinations amid delta surge. *The New York Times*, 2021. https://www.washingtonpost.com/politics/growing-number-of-republicans-urge-vaccinations-amid-delta-surge/2021/07/20/52a06e9c-e999-11eb-8950-d73b3e93ff7f_story.html.

6. Lydia Saad. More in u.s. vaccinated after delta surge, fda decision. *Gallup*, 2021. https://news.gallup.com/poll/355073/vaccinated-delta-surge-fda-decision.aspx.

7. Kate Larsen. Reports of menstrual cycle changes after covid vaccine highlight issues with clinical trials. *ABC7 News*, 2021. https://abc7news.com/covid-vaccine-menstrual-cycle-clinical-trials-and-side-effects-women/10557707/.

8. Anika Nayak. How the covid-19 vaccine may affect your period (and what to do about it). *HuffPost*, 2021. https://www.huffpost.com/entry/covid-19-vaccine-affect-period_l_6086d4f9e4b09a22a4456632.

9. Jennifer Abbasi. Widespread misinformation about infertility continues to create covid-19 vaccine hesitancy. *JAMA*, 327(11):1013–1015, 2022.

10. Everett M. Rogers. *Diffusion of Innovations, 5th Edition*. Simon and Schuster, 2003.

11. Everett M. Rogers and F. Floyd Shoemaker. *Communication of Innovations: A Cross-Cultural Approach*. Free Press, 1971.

12. USA Facts. Us coronavirus vaccine tracker, 2023. https://usafacts.org/visualizations/covid-vaccine-tracker-states/.
