# Supplementary material for: Measuring vaccination coverage and concerns of vaccine holdouts from web search logs
Source: Nat Commun. 2024 Aug 1;15:6496. doi: 10.1038/s41467-024-50614-4 (PMC11294546; doi:10.1038/s41467-024-50614-4)
Supplement: Supplementary file 3 — Reporting Summary [file 41467_2024_50614_MOESM3_ESM.pdf]

nature portfolio

Corresponding author(s): Eric Horvitz

Last updated by author(s): 2024/04/17

Reporting Summary

Nature Portfolio wishes to improve the reproducibility of the work that we publish. This form provides structure for consistency and transparency in reporting. For further information on Nature Portfolio policies, see our Editorial Policies and the Editorial Policy Checklist.

Statistics

For all statistical analyses, confirm that the following items are present in the figure legend, table legend, main text, or Methods section.

n/a	Confirmed	
☐	☒	The exact sample size (*n*) for each experimental group/condition, given as a discrete number and unit of measurement
☐	☒	A statement on whether measurements were taken from distinct samples or whether the same sample was measured repeatedly
☐	☒	The statistical test(s) used AND whether they are one- or two-sided<br>*Only common tests should be described solely by name; describe more complex techniques in the Methods section.*
☐	☒	A description of all covariates tested
☐	☒	A description of any assumptions or corrections, such as tests of normality and adjustment for multiple comparisons
☐	☒	A full description of the statistical parameters including central tendency (e.g. means) or other basic estimates (e.g. regression coefficient) AND variation (e.g. standard deviation) or associated estimates of uncertainty (e.g. confidence intervals)
☐	☒	For null hypothesis testing, the test statistic (e.g. *F*, *t*, *r*) with confidence intervals, effect sizes, degrees of freedom and *P* value noted<br>*Give P values as exact values whenever suitable.*
☒	☐	For Bayesian analysis, information on the choice of priors and Markov chain Monte Carlo settings
☒	☐	For hierarchical and complex designs, identification of the appropriate level for tests and full reporting of outcomes
☐	☒	Estimates of effect sizes (e.g. Cohen's *d*, Pearson's *r*), indicating how they were calculated

Our web collection on statistics for biologists contains articles on many of the points above.

Software and code

Policy information about availability of computer code

Data collection	We use existing data for almost all of our analyses, so we are not collecting new data. Our main data source is anonymized search logs from Bing. The Bing data we use consists of individual queries made by users, where for each query, we have information including the text of the query, an anonymized ID of the user, the timestamp, the estimated geolocation (ZIP code, county, and state), and the set of URLs clicked on, if any. We also use public data sources, including vaccination rates from the Centers for Disease Control and Prevention (CDC), ZIP-level vaccination rates from local public health departments, demographic data from the US Census, county-level data from the 2020 US Presidential Election, labels of news sites from Newsguard, and Google search trends. Data analyses were approved by the Bing product team and Microsoft IRB. See Methods Section M1 for details.<br><br>The only data we collect are annotations on Amazon Mechanical Turk (AMT) to label URLs and queries for vaccine intent. We designed our task on AMT's interface and include screenshots of our task in the manuscript (Figure 5). We did not use software to collect annotations. Our annotation task was also approved by Microsoft IRB, and annotators on AMT are required to sign a consent form before completing our tasks. See Methods Section M2.2 for details.
Data analysis	We apply our vaccine intent classifier to anonymized Bing search logs and identify 7.45 million Bing users who have expressed vaccine intent. Then, we 1) compute summary statistics of vaccine intent rates per ZIP code, county, and state, 2) compare the aggregated search interests of a subset of these users (212,283 "vaccine holdouts" and 212,283 "early adopters"). See Methods Sections M3 and M4 for details.<br><br>All code is available at https://github.com/microsoft/vaccine_search_study.

For manuscripts utilizing custom algorithms or software that are central to the research but not yet described in published literature, software must be made available to editors and reviewers. We strongly encourage code deposition in a community repository (e.g. GitHub). See the Nature Portfolio guidelines for submitting code & software for further information.

Data

Policy information about availability of data

All manuscripts must include a data availability statement. This statement should provide the following information, where applicable:

- Accession codes, unique identifiers, or web links for publicly available datasets
- A description of any restrictions on data availability
- For clinical datasets or third party data, please ensure that the statement adheres to our policy

Our vaccine intent estimates and ontology of vaccine concerns are publicly available at https://github.com/microsoft/vaccine_search_study. Aside from Bing search logs, all of the data sources that we use are publicly available online. The CDC data, ZIP-level vaccination rates, US Census data, and Google search trends can be directly downloaded, and the elections data and Newsguard data can be purchased at the references provided in Section M1.

Research involving human participants, their data, or biological material

Policy information about studies with human participants or human data. See also policy information about sex, gender (identity/presentation), and sexual orientation and race, ethnicity and racism.

Reporting on sex and gender	We do not collect or analyze individual-level data on sex or gender. In order to analyze demographic trends in estimated vaccine intent rates, we use percent female (sex) per ZIP code tabulation area (ZCTA), as reported in the US Census' 2020 5-year American Community Survey.
Reporting on race, ethnicity, or other socially relevant groupings	We do not collect or analyze individual-level data on race or ethnicity. In order to analyze demographic trends in estimated vaccine intent rates, we use percent White, percent Black, percent Asian, and percent Hispanic/Latino per ZCTA, as reported in the US Census' 2020 5-year American Community Survey.
Population characteristics	We also use population size, percent with Bachelor degree or higher, median income, population per square meter, and percent of different age groups per ZCTA from the US Census' 2020 5-year American Community Survey. We use county-level data from the 2020 US Presidential Election.
Recruitment	We use existing data for almost all of our analyses, so we are not recruiting participants. However, we carefully address potential biases in our approach in Section M3. First, we show that bias can be decomposed into two sources: bias from non-uniform Bing coverage and bias from non-uniform classifier performance. Then, we estimate Bing coverage rates per ZCTA and correct for these non-uniform rates. Finally, we evaluate classifier performance and show that it achieves uniformly strong performance (AUCs, true positive rates, and false positive rates) across regions.<br><br>For our annotation task, we recruit annotators by posting the task on Amazon Mechanical Turk (AMT). While annotators may not be a random sample of the population, we do not expect that their labels are biased by their identities, since the task is straightforward (whether a URL/query indicates vaccine intent). To further mitigate annotator bias/noise, we acquire three annotations per URL/query and require strong agreement (at least three annotators out of three or four) to assign a positive label.
Ethics oversight	For data analyses: Microsoft IRB office, privacy officers from Microsoft Research and Bing product team<br>For Amazon Mechanical Turk task: Microsoft IRB office, consent form<br>For data and code release: Microsoft Licensing and Legal, Privacy, Responsible AI, and Security

Note that full information on the approval of the study protocol must also be provided in the manuscript.

Field-specific reporting

Please select the one below that is the best fit for your research. If you are not sure, read the appropriate sections before making your selection.

☐ Life sciences ☒ Behavioural & social sciences ☐ Ecological, evolutionary & environmental sciences

For a reference copy of the document with all sections, see nature.com/documents/nr-reporting-summary-flat.pdf

Behavioural & social sciences study design

All studies must disclose on these points even when the disclosure is negative.

Study description	We analyze large-scale anonymized search logs from Bing and develop machine learning methods to extract vaccine-related behaviors. Analyses are quantitative and computational.
Research sample	Our work leverages billions of anonymized search logs from Bing. Bing is the second largest search engine worldwide and in the US, with a US market share of around 6% on all platforms and around 11% on desktop. Despite having non-uniform coverage across the US, Bing has enough penetration across the country that we can estimate representative samples after applying inverse proportional weighting (Section M3.2). The Bing data we use consist of individual queries made by users, where for each query, we have information including the text of the query, an anonymized ID of the user, the timestamp, the estimated geolocation (ZIP code,

	county, and state), and the set of URLs clicked on, if any. Since our work is motivated by insufficient vaccine data and vaccine concerns in the US, we limit our study to search logs in the US market. However, the methods we introduce could be extended to study vaccination rates and vaccine concerns in other languages and countries. We apply our vaccine intent classifier (Section M2) to Bing search logs from February 1 to August 31, 2021. February 2021 was the earliest that we could study following data protection guidelines, which allow us to store and analyze search logs up to 18 months in the past. We end in August 2021, since the FDA approved booster shots in September and our method is not designed to disambiguate between vaccine seeking for the primary series versus boosters.
Sampling strategy	As described above, our study uses Bing search logs in the US from February to August 2021. For each month, we include "active" Bing users, i.e., those who issued at least 30 queries in the month, so that we can reliably assign a user to a location (ZIP code, county, and state) based on their mode location among their queries in the month. Using our vaccine intent classifier, we identify 7.45 million active Bing users in the US who expressed vaccine intent between February and August 2021.<br><br>In later analyses, we focus on a subset of these users. We define early adopters as those who showed their first vaccine intent before May (i.e., between February 1 and April 30, 2021) and vaccine holdouts as those who waited until July to show their first vaccine intent (i.e., between July 1 and August 31, 2021). We choose these cutoffs since all US residents aged 16 and older were eligible for the vaccine by April 19, so those who waited until July to seek the vaccine were holding out. Furthermore, to improve our ability to detect true holdouts, we require holdouts and early adopters to be active (i.e., issued at least 30 queries) in every month during the study period, since if users were not active on Bing before July or August, their apparent lack of vaccine intent could be explained simply by low Bing usage during the earlier months. Finally, we did not consider as holdouts those who never showed vaccine intent during our study period, since those users may have gotten their vaccine in ways that are not visible via search data, e.g., a walk-in appointment. In comparison, individuals who did not show their first vaccine intent until July 2021 likely did not receive the vaccine before. To reduce potential confounding in comparisons, we match vaccine holdouts to early adopters from the same county and with a similar query count (Section M4.1). This results in 212,283 matched pairs.
Data collection	The only data we collect are annotations on Amazon Mechanical Turk (AMT) to label URLs and queries for vaccine intent. We designed our task on AMT's interface and include screenshots of our task in the manuscript (Figure 5). We did not use software to collect annotations. Our annotation task was also approved by Microsoft IRB, and annotators on AMT are required to sign a consent form before completing our tasks. See Methods Section M2.2 for details.
Timing	We study Bing search logs from February to August 2021, for the reasons stated above ("Research sample").
Data exclusions	We focus on active Bing users in the US, for the reasons stated above ("Sampling strategy"). Using our vaccine intent classifier, we identify 7.45 million active Bing users in the US who expressed vaccine intent between February and August 2021. After filtering on vaccine intent timing, being active in every month during the study period, and matching vaccine holdouts and early adopters, we are left with 212,283 matched pairs.
Non-participation	We did not recruit participants on Bing. On Amazon Mechanical Turk, we can only see annotations from workers who choose to accept our task, but cannot track workers who decline our task.
Randomization	To compare click rates (e.g., holdouts vs early adopters), we use bootstrapped sampling of URL clicks to obtain 95% confidence intervals (Section M5.2). To compare the average vaccine intent rates of ZCTA sets (e.g., top income quartile vs bottom income quartile), we use bootstrapped sampling of ZCTAs to obtain 95% confidence intervals (Section M5.1). In both cases, we repeatedly resample with replacement to obtain 1000 bootstrapped samples. Point estimates are provided using the full data (not sampled).<br><br>We also employ randomization to assess the performance of our graph neural networks (GNNs). To account for randomness from model training and data splitting, we run 10 random trials for every GNN/state where in each trial, we re-split the URL labels into train, validation, and test sets, retrain the model on the train set (stopping based on the validation loss), and re-evaluate the model's final performance on the test set. AUCs, true positive rates, and false positive rates are evaluated per trial, and we report their means and standard deviations.

Reporting for specific materials, systems and methods

We require information from authors about some types of materials, experimental systems and methods used in many studies. Here, indicate whether each material, system or method listed is relevant to your study. If you are not sure if a list item applies to your research, read the appropriate section before selecting a response.

Materials & experimental systems

n/a	Involved in the study
☒	☐ Antibodies
☒	☐ Eukaryotic cell lines
☒	☐ Palaeontology and archaeology
☒	☐ Animals and other organisms
☒	☐ Clinical data
☒	☐ Dual use research of concern
☒	☐ Plants

Methods

n/a	Involved in the study
☒	☐ ChIP-seq
☒	☐ Flow cytometry
☒	☐ MRI-based neuroimaging
